# Using neurolipidomics to identify phospholipid mediators of synaptic (dys)function in Alzheimer's Disease

**DOI:** 10.3389/fphys.2013.00168

**Published:** 2013-07-16

**Authors:** Steffany A. L. Bennett, Nicolas Valenzuela, Hongbin Xu, Bettina Franko, Stephen Fai, Daniel Figeys

**Affiliations:** ^1^Ottawa Institute of Systems BiologyOttawa, ON, Canada; ^2^Neural Regeneration Laboratory, Department of Biochemistry, Microbiology, and Immunology, University of OttawaOttawa, ON, Canada; ^3^CIHR Training Program in Neurodegenerative Lipidomics, Department of Biochemistry, Microbiology, and Immunology, University of OttawaOttawa, ON, Canada; ^4^Carleton Immersive Media Studio, Azrieli School of Architecture and Urbanism, Carleton UniversityOttawa, ON, Canada

**Keywords:** neurolipidomics, phospholipid, Alzheimer's Disease, super resolution nanoscopy, lipidomics, mass spectrometry, amyloid-beta, synaptotoxicity

## Abstract

Not all of the mysteries of life lie in our genetic code. Some can be found buried in our membranes. These shells of fat, sculpted in the central nervous system into the cellular (and subcellular) boundaries of neurons and glia, are themselves complex systems of information. The diversity of neural phospholipids, coupled with their chameleon-like capacity to transmute into bioactive molecules, provides a vast repertoire of immediate response second messengers. The effects of compositional changes on synaptic function have only begun to be appreciated. Here, we mined 29 neurolipidomic datasets for changes in neuronal membrane phospholipid metabolism in Alzheimer's Disease (AD). Three overarching metabolic disturbances were detected. We found that an increase in the hydrolysis of platelet activating factor precursors and ethanolamine-containing plasmalogens, coupled with a failure to regenerate relatively rare alkyl-acyl and alkenyl-acyl structural phospholipids, correlated with disease severity. Accumulation of specific bioactive metabolites [i.e., PC(*O*-16:0/2:0) and PE(*P*-16:0/0:0)] was associated with aggravating tau pathology, enhancing vesicular release, and signaling neuronal loss. Finally, depletion of PI(16:0/20:4), PI(16:0/22:6), and PI(18:0/22:6) was implicated in accelerating Aβ_42_ biogenesis. Our analysis further suggested that converging disruptions in platelet activating factor, plasmalogen, phosphoinositol, phosphoethanolamine (PE), and docosahexaenoic acid metabolism may contribute mechanistically to catastrophic vesicular depletion, impaired receptor trafficking, and morphological dendritic deformation. Together, this analysis supports an emerging hypothesis that aberrant phospholipid metabolism may be one of multiple critical determinants required for Alzheimer disease conversion.

## Neurolipidomics: cataloging functional diversity in membrane biology

**Figure 1:**
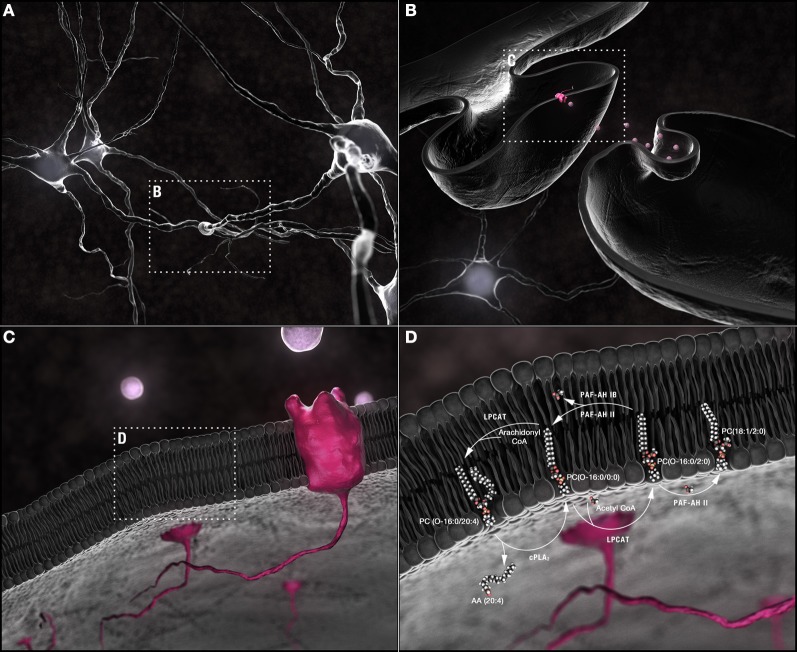
**Synaptic membrane remodeling. (A)** Chemical neurotransmission occurs at the synapse. **(B)** Neurotransmitters, released into the synaptic cleft following fusion of vesicular with pre-synaptic plasma membranes, interact with receptors shuttled along the plasma membrane of post-synaptic dendritic spines. **(C)** Phospholipids are the most abundant components of neuronal membranes. **(D)** Dynamic phospholipid remodeling enables the rapid alterations in membrane form and function required for synaptic transmission while maintaining critical lipid composition essential for synaptic structural integrity. The Land's cycle is depicted. Hydrolysis of the *sn*-2 arachidonyl chain of a 1-*O*-ether-linked choline-containing glycerophospholipid by cPLA_2_ releases AA (20:4). The residual *lyso*-PAF backbone can be remodeled by LPCAT1 at the plasma membrane into the powerful PAF family of neuromodulators or reconstructed back into a 1-*O*-alkyl-linked structural lipid depending upon whether an acetyl group or a long chain fatty acid, primed by the actions of acyl-Co synthetase, is used as a substrate. In turn, the *sn*-2 acetyl group released by PAF-AH during conversion of PAFs back to *lyso*-PAFs can be passed to an ester-linked *lyso*phosphatidylcholine, a sphingolipid, or another ether-linked *lyso*-PAF with either the same or different *sn*-1 chains. PAF-AH 1b does not have transferase activity; PAFAH II does. This single pathway alone can generate a plethora of different phospholipids through the simple exchange of constituent hydrocarbon chains. Abbreviations: AA, arachidonic acid; cPLA_2_, cytoplasmic PLA_2_; CoA, coenzyme A; LPCAT1, *lyso*phosphatidylcholine acyltransferase 1; *lyso*-PAF, *lyso*-platelet activating factor or 1-*O*-alkyl *lyso*glycerophosphocholine; PAF, platelet activating factor or 1-*O*-alkyl-2-acetyl-glycerophosphocholine; PAF-AH, PAF-acetylhydrolase.

**Figure 2:**
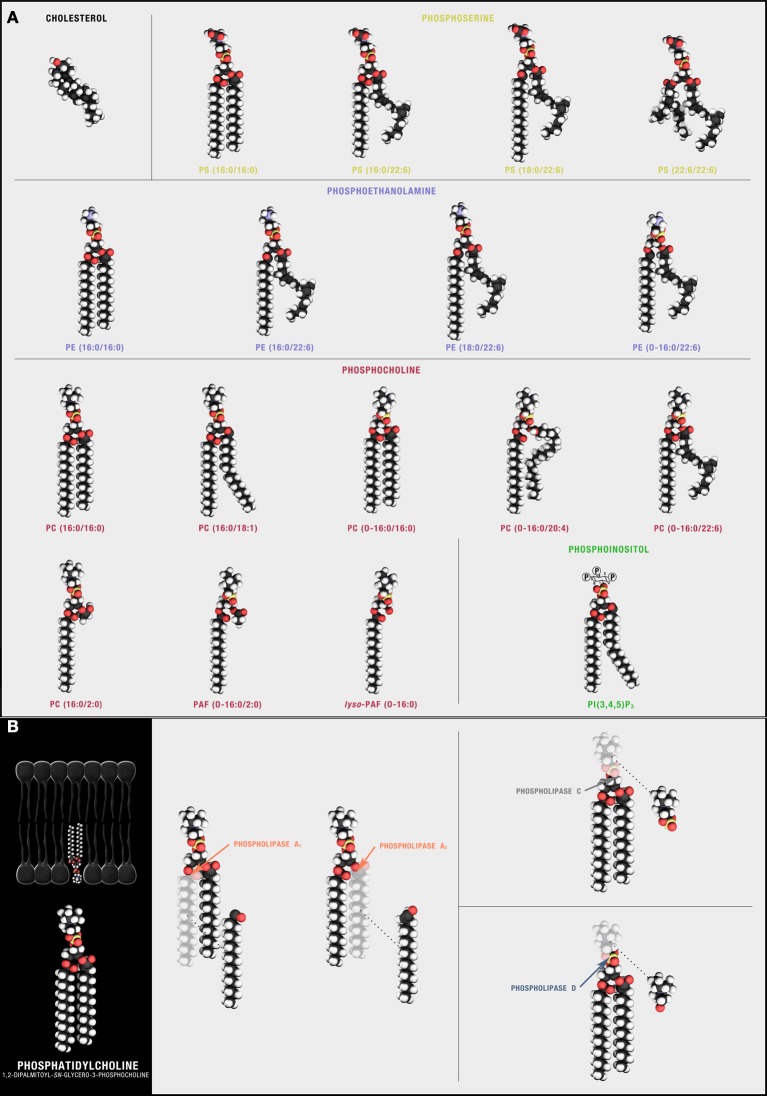
**Synaptic phospholipids (and cholesterol). (A)** Structural membrane phospholipids are derivatives of *sn*-glycero-3-phosphoric acid with a 1-*O*-acyl, a 1-*O*-alkyl (ether-linked plasmanyl), or a 1-*O*-alkyl-1′-enyl (vinyl ether-linked plasmenyl) carbon chain, a long-chain fatty acid esterified to the *sn*-2 position, and a polar headgroup composed of a nitrogenous base, a glycerol, or an inositol unit modifying phosphoric acid at the *sn*-3 position. The polar head group defines different phospholipid classes with 1-*O*-acyl linked PS, phosphatidylserine; PA, phosphatidic acid; PI, phosphatidylinositol; PE, phosphatidylethanolamine; and PC, phosphatidylcholine predominant in neuronal plasma membranes. The free diffusion of these species through the lipid bilayer is, in part, influenced by cholesterol, a sterol defined by a four fused-ring core and an alkyl side-chain that preferentially solvates with some but not all *sn*-1 and *sn*-2 phospholipid side chains. (**B**) Phospholipids can be hydrolyzed by different families of PLAs. Cleavage sites are depicted using PC(16:0/16:0) as the target substrate.

The field of neurolipidomics seeks to understand how dynamic changes in membrane composition regulate brain cell function. Here, we mined 29 different neurolipidomic datasets generated by 11 independent laboratories for critical changes in neural membrane phospholipid metabolism (Tables S1–S4). We then asked how these changes might mechanistically contribute to synaptic dysfunction in Alzheimer's Disease (AD). Neuronal membranes are enriched in sterols, sphingolipids, glycerolipids, and phospholipids (Figure 1A). Phospholipids are the most abundant. Their assembly into lipid bilayers, with polar head groups aligning at aqueous interfaces and hydrophobic carbon chains buried within, produces the semi-permeable barriers of cellular (and subcellular) membranes (Figures 1B,C). These bilayers are often conceptualized as undulating fields of identical molecules (Figure 1C). In fact, they are complex matrices of several hundred molecularly distinct species (Figure 2A). Composition is in constant flux. Carbon chains and defining polar head groups are dynamically exchanged by activated phospholipases and *lyso*phospholipid transferases in response to environmental stimuli (Figures 1D, 2B). Until recently, this diversity has remained largely under-appreciated and functional significance unexplored. However, for the first time, significant technological advances in high performance liquid chromatography (LC), electrospray ionization (ESI), and matrix-assisted laser desorption ionization (MALDI) mass spectrometry (MS) are enabling membrane composition to be profiled comprehensively at the molecular level^1^. Coupled with subcellular fractionation and careful consideration of extraction protocols that enrich for different phospholipid families, species that vary by only one double bond, a single methylene group, or carbon chain linkage can now be quantified directly in synaptic preparations. Further, new high-resolution optical single molecule tracking approaches, notably fluorescence correlation spectroscopy (FCS) coupled with the stimulated emission depletion fluorescence nanoscopy (STED) and fluorescence lifetime imaging microscopy (FLIM), are facilitating, again for the first time, the study of functional interactions specific to lipid microdomains directly in living cells (Eggeling et al., 2009; Kusumi et al., 2010; Sahl et al., 2010; Mueller et al., 2013). Basic unitary conceptions are being challenged. Diacylglycerol (DAG), commonly conceived by cell biologists as a single lipid second messenger, is now recognized to be a family of over 50 structurally distinct isoforms (Callender et al., 2007) each controlling different cellular processes (Deacon et al., 2002).

## Exploring a “lipid-centric” view of synaptic function and dysfunction in AD

### Conversion from a pre-symptomatic to a symptomatic state in AD requires multiple metabolic disruptions

Two central pathologies define AD: (1) intraneuronal accumulation of neurofibrillary tangles composed of hyperphosphorylated tau and (2) aberrant processing of the amyloid precursor protein (APP) to smaller, toxic amyloid β (Aβ) fragments. The most damaging is Aβ_42_. Accumulation is gradual with assembly of soluble Aβ_42_ oligomers impairing synaptic function and signaling neuronal loss (Cleary et al., 2005; Palop and Mucke, 2010; Benilova et al., 2012). The “amyloid cascade hypothesis” defines these events as the root cause of AD (Hardy and Selkoe, 2002; Palop and Mucke, 2010; Benilova et al., 2012). Yet new data suggests that these driving Aβ and tau pathologies likely represent only two of multiple determinants required for AD conversion. This refinement is based on evidence that Aβ_42_ accumulation (at AD load levels) can be detected in both cognitively “normal” elderly and humanized mouse models with little learning and memory impairment (Snowdon, 2003; Zahs and Ashe, 2010). These observations have prompted a “re-imagining” of the amyloid hypothesis (Herrup, 2010; Kuller and Lopez, 2011; Nelson et al., 2011). Here, the amyloid deposition cycle, aggravated by chronic neuroinflammation, triggers a critical “change in state” (Herrup, 2010). This “change of state” is envisioned as a convergence of metabolic disruptions resulting in “a new ‘normal’ biology primed toward neurodegeneration and dementia” (Herrup, 2010).

### Pathological membrane remodeling may represent a critical metabolic disruption required for AD conversion

Our overarching hypothesis is that multiple aberrations in phospholipid metabolism are required for transition from pre-symptomatic to symptomatic AD. Here, we seek to identify some of these additional critical disruptions by mining existing neurolipidomic datasets. Previous work has correlated the extent of membrane phosphocholine (PC) and phosphoethanolamine (PE) breakdown with severity of dementia and psychosis in AD patients (Klein, 2000; Sweet et al., 2002). Moreover, soluble oligomeric Aβ_42_ neurotoxicity is signaled, in part, by enhanced metabolism of ether linked structural phospholipids (Sanchez-Mejia et al., 2008; Ryan et al., 2009). Targeting upstream remodeling or downstream signaling of key metabolites can protect human and murine neurons from Aβ_42_ *in vitro* and improve behavioral indices of learning and memory *in vivo* (Kriem et al., 2005; Sanchez-Mejia et al., 2008; Ryan et al., 2009). There is also compelling evidence to indicate that the fatty acid substrates available for phospholipid biosynthesis are altered over the course of AD (Table S5). For example, using an unbiased neurolipidomics approach, non-esterfied monounsaturated fatty acid (MUFA) levels have been shown to increase in AD brain whereas polyunsaturated (PUFA) levels generally decrease (Lukiw, 2009; Astarita et al., 2010, 2011). These changes are attributed to increased expression of three of the rate-limiting enzymes in MUFA biosynthesis, stearoyl-CoA desaturase-1 (SCD-1) SCD-5a and SCD-5b (Astarita et al., 2011). Enhanced activity is thought to compensate for decreasing bioavailability of PUFA substrates highlighting the molecular interdependency of lipid metabolic defects that occur over the course of AD (Astarita et al., 2011).

These disruptions may confer AD risk. Recent genome wide association studies (GWAS) have identified variants in sortilin-related receptor (Rogaeva et al., 2007), clusterin (Harold et al., 2009; Lambert et al., 2009; Jun et al., 2010), bridging integrator 1 (Seshadri et al., 2010), ATP-binding cassette sub-family A member 7 (Hollingworth et al., 2006), and phosphatidylinositol binding clathrin assembly protein (Harold et al., 2009; Jun et al., 2010) genes as AD risk factors. Clearly, these genes do not act through the same biochemical pathways yet each shares in common a regulation of one or more aspects of neural phospholipid (and in some cases cholesterol) metabolism with the canonical ApoE risk allele (Strittmatter et al., 1993). Excitingly, there is also evidence that, once aberrant patterns in phospholipid metabolism are identified, intervention may be possible. Enhancing apolipoprotein E (ApoE)-dependent trafficking of PUFAs from neurons to glia in APP/presenilin 1 (PS1) transgenic mice changes the phospholipid composition of synaptosomes, increases Aβ_42_ clearance, and reverses learning and memory impairment (Igbavboa et al., 2002; Cramer et al., 2012). Conversely, inhibition or genetic ablation of phospholipase D_2_ (PLD_2_) (Figure 2B), the group IV isoform of PLA_2_ (cPLA_2_) (Figure 2B), or synaptojamin, the primary phosphoinositide PI(4,5)P_2_ phosphatase also confers synaptic protection, reduces Aβ_42_ biosynthesis, and rescues memory deficits in APP transgenics (Berman et al., 2008; Sanchez-Mejia et al., 2008; Oliveira et al., 2010).

## Interpreting synaptic glycerophospholipidome datasets

**Figure 3:**
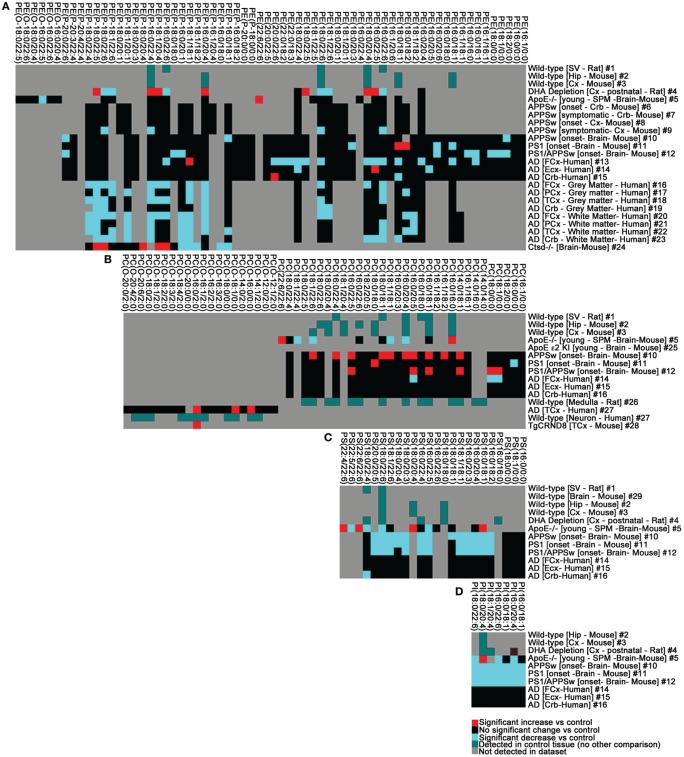
**The phospholipid landscape is altered in AD and mouse models of AD or AD risk. (A)** PE, **(B)** PC, **(C)** PS, **(D)** PI. Heat map display of significant interactions (*p* < 0.05) statistically validated by the authors of the following datasets: #1 (Takamori et al., 2006), #2–3 (Axelsen and Murphy, 2010), #4 (Brand et al., 2010), #5 (Igbavboa et al., 2002), #6–9 (Han et al., 2001), #10–15 (Chan et al., 2012), #16–23 (Han et al., 2001), #24 (Mutka et al., 2010), #25 (Sharman et al., 2010); #26 (Lohmann et al., 2010), #27–28 (Ryan et al., 2009), #29 (Eberlin et al., 2010). The color scheme reflects statistical changes in ApoE^−/−^, ApoE ε2, ε3, ε4 humanized knock-in (KI), cathepsinD (Ctsd)^−/−^, APPSw transgenic, PS1 transgenic, PS1/APPSw double transgenic, TgCRND8 transgenic mice, and post-mortem human AD brain tissue relative to cognate controls as published. Where comparisons were not made with disease or experimental conditions, color (green) simply reflects detection in datasets. Where published datasets identified lipids by total number of carbons and degree of unsaturation, assignment of *sn*-1 and *sn*-2 chains in this study was performed as indicated in Tables S1–S4 (footnote#1). Tissue abbreviations: Cx, Cortex; Crb, cerebellum; ECx, Entorhinal Cortex; FCx, Frontal Cortex/Prefrontal Cortex; Hip, Hippocampus; PCx, Parietal Cortex; SPM, Synaptosomal membranes; SV, Synaptic Vesicle. All other conditions are as described in Tables S1–S4.

**Figure 4:**
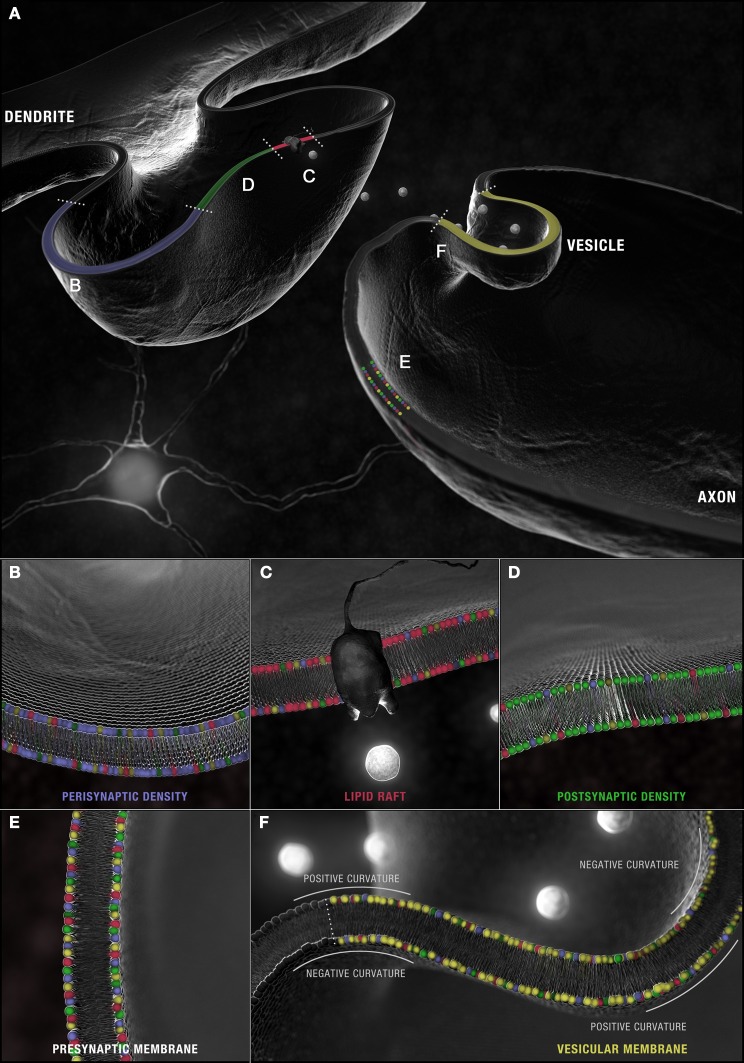
**Synaptic microdomains exhibit different critical structural phospholipid compositions that are remodeled over the course of neurotransmission. (A)** In a simplified membrane-centric model of excitatory neurotransmission, neurotransmitter released into the synaptic cleft from vesicles by the pre-synaptic neuron, diffuses across the intersynaptic space, to activate integral membrane receptors present along dendritic spine microdomains. In the post-synaptic neuron, receptors (and other necessary signaling components) can be shuttled between **(B)** peri- and **(D)** post-synaptic densities in **(C)** lipid rafts. An incoming axon potential at the axon terminal can trigger the fusion of **(F)** synaptic vesicles along the active zone within **(E)** pre-synaptic densities and the release of neurotransmitter. Each domain is defined by a distinct profile of constituent PC (red), PS (yellow), PE (blue) and PI (green) with critical compositions required for structural integrity maintained constant by this dynamic remodeling.

We used a *post-hoc* neurolipidomics approach to identify potential phospholipid determinants of AD cognitive impairment. We first collated the findings of 29 published neurolipidomic datasets generated using multiple methodologies, technologies and human and murine samples (Tables S1–S4). If not explicitly identified by the studies' authors, we used the online bioinformatics tool VaLid to predict *sn*-1 and *sn*-2 carbon chain stereospecificity (Blanchard et al., 2013). Results were combined, heat-mapped, and mined for underlying patterns (Figure 3). This analysis required that we set aside two of fundamental assumptions commonly applied to the interpretation of genomic and proteomic datasets. First, we postulated that critical compositions of synaptic lipids are likely not the ones altered early in disease etiology but rather the species that exhibit a catastrophic depletion in late-stage disease. Unlike mRNA and protein, individual phospholipids do not have their own synaptic half-lives and cannot be tracked as single entities from biogenesis to catabolism. Rather, following linkage determination in the endoplasmic reticulum and transport to synaptosomal membranes, their defining *sn*-1 and *sn*-2 carbon chains (depending on linkage to the phosphoglyceride backbone) and, to some extent, their polar head groups are passed between species. Each of the free fatty acids released by PLA_1_ or PLA_2_ hydrolysis (Figure 2B) has its own synaptic half-life, ranging from 10 days for palmitic acid (16:0) to 60 days for AA (20:4) (Ando et al., 2002). This metabolic consubstantiality, wherein critical chemical compositions are maintained through dynamic remodeling of multiple species, enables the rapid changes in membrane curvature (and fusion) required for neurotransmission while ensuring synaptic structural integrity (Figures 4A–F). We argue that any quantifiable shift in these critical compositions likely indicates that a catastrophic “change in state” has already occurred (i.e., evidence of a membrane biology now “primed toward neurodegeneration and dementia”). Second, we postulated that variations between datasets in the composition of smaller glycerophospholipid intermediates (i.e., the *lyso*phospholipids, free fatty acids, and their downstream metabolites) (Figure 1C) are not “noise” but likely (1) snapshots of the structural species being actively mobilized to maintain critical structural membrane compositions at time of extraction and (2) identify the neuroactive phospholipid signaling molecules that transiently accumulate in different disease states. We argue that these changes will emerge earlier than catastrophic alterations in overall structural phospholipid composition. Taken together, we maintain that the interpretation of lipidomic datasets depends less upon a determination of absolute phospholipid levels (which will vary considerably from laboratory to laboratory and methodology to methodology) than upon identification of the patterns in lipid composition that are altered over the course of disease progression (Brown and Murphy, 2009). Here, we present three such patterns in membrane metabolism identified through analysis of 29 independent datasets predicted to signal synaptic dysfunction in AD.

## Pattern 1: evidence for synaptic poisoning: a primary role for ether-linked phospholipids in mediating AD vesicular depletion

### Synaptic microdomains exhibit different phospholipid profiles

Enrichment in PC, PE, PI, PS, specifically isoforms with (16:0) and (18:1) carbon chains at both their *sn*-1 and *sn*-2 positions, is detected in synaptosomal membranes prepared from healthy mouse, rat, and human brain (Tables S1–S4)^2^. Given the dietary abundance of palmitic acid and oleic acid, this pattern is, of course, expected. Yet there is an intriguing molecular specificity that distinguishes different synaptic microdomains (Figure 4A). In healthy murine tissue, the relative abundance of PCs and 1-*O*-alkyl-PC isoforms (16:0/18:1) and (18:0/18:1) are higher in pre- and post-synaptic densities relative to lipid raft domains (Igbavboa et al., 2002; Martin et al., 2010) (Figures 4C–E). In synaptic vesicles, PC(16:0/18:1)^3^ and, presumably PC(*O*-16:0/18:1), predominate over PC(18:0/18:1) and PC(*O*-18:0/18:1) (Takamori et al., 2006) (Figure 4F). Vesicular membranes (and synaptic lipid rafts) are further defined by enrichment in diacyl-PCs with fully saturated palmitic acid carbon chains [i.e., PC(16:0/16:0)] but only a small percentage of the more elastic PS (and PI) (16:0/16:0) species compared to non-raft domains (Takamori et al., 2006; Martin et al., 2010). Finally, fewer acyl-linked PC, PE, and PS species with docosahexaenoic acid (DHA, 22:6) at their *sn*-2 positions are found in isolated synaptic vesicles relative to other synaptosomal microdomains and brain tissue (Williams et al., 2000; Han et al., 2001; Takamori et al., 2006; Wurtman et al., 2009; Axelsen and Murphy, 2010; Martin et al., 2010).

### PC(16:0/16:0), PC(O-16:0/20:4), PC(O-16:0/22:6), PE(P-18:0/20:4), and PE(P-16:0/22:6) represent critical vesicular phospholipid compositions

These restrictions are surprisingly linkage- and position-specific. Unlike acyl-linked PCs and PEs, 1-*O*-alkyl-linked species (PAF precursors) and ethanolamine-containing 1-*P*-alkenyl plasmalogens (PlsEtns) in brain frequently exhibit either AA (20:4) or DHA (22:6) in addition to oleic acid (18:1) at their *sn*-2 positions (Igbavboa et al., 2002; Takamori et al., 2006; Bruno et al., 2007; Martin et al., 2010). Moreover, in PAF precursors, fully saturated *sn*-1 ether-linked (16:0) chains dominate [PC(*O*-16:0/20:4) or PC(*O*-16:0/22:6)]. By contrast, PlsEtns display a positional preference for either vinyl ether-linked stearic acid (18:0) at their *sn*-1 and acyl-linked AA (20:4) at their *sn*-2 positions^4^ or vinyl ether-linked (16:0) chains at their *sn*-1 and acyl-linked DHA (22:6) at their *sn*-2 positions^5^ (Han et al., 2001; Igbavboa et al., 2002; Takamori et al., 2006; Ryan et al., 2009; Axelsen and Murphy, 2010; Brand et al., 2010; Eberlin et al., 2010; Lohmann et al., 2010; Sharman et al., 2010). Together, these profiles suggest a higher requirement for 16 carbon chains at both the *sn*-1 and *sn*-2 positions, a predominance of diacyl PC(16:0/16:0), an enrichment of PC(*O*-16:0/20:4) and PC(*O*-16:0/22:6) PAF precursors, and an abundance of the PlsEtns PE(P-18:0/20:4) and PE(P-16:0/22:6) in vesicular membranes of “normal” synapses.

### Membrane remodeling required for neurotransmitter release is regulated, in part, by the substrate specificities of synaptic phospholipases

Vesicle diameter, measured by cryo-electron microscopy, ranges between 30 and 60 nm, with an average diameter of 42 nm (Takamori et al., 2006; Castorph et al., 2010). A single phospholipid occupies a space of ~65 Å^2^ (0.65 nm^2^) (Takamori et al., 2006; Castorph et al., 2010). With phospholipid composition calculated as 50–75% of the total lipid content, each 42 nm diameter vesicle is estimated to contain around 7000 individual species (McMahon and Gallop, 2005; Takamori et al., 2006). Structure, in part, dictates topography. The extreme curvature of vesicular membranes places more phospholipids in the outer and fewer in the inner leaflet to balance the spatially larger exterior positive curvature with the spatially smaller interior negative curvature (McMahon and Gallop, 2005; Takamori et al., 2006) (Figure 4F). Phospholipid identity within these leaflets must also reconcile the need to stabilize vesicular structure with the requirement to rapidly reverse membrane curvature and fuse with the plasma membrane on demand (Figure 4F). In a protein-centric view, these dynamics are conceptually better suited to pliant fusogenic phospholipids whose physicochemical natures are “released” once the structural constraints enforced by integral membrane proteins are relaxed (McMahon and Gallop, 2005). Such a model makes the predominance of saturated diacyl PC(16:0/16:0) lipids in isolated synaptic vesicles somewhat surprising (Takamori et al., 2006). Reconstitution studies indicate that the cylindrical geometries of dipalmitoyl-PCs are more suited to the generation of planar membranes or, in presence of water, to the formation of vesicles with a longer-lived stable structure under negative tension (McMahon and Gallop, 2005; Shinoda et al., 2010). The enrichment of PC(16:0/16:0) would therefore be expected to render synaptic vesicles more fusion-resistant yet these neurolipidomic profiles are supported by small angle neutron scattering analysis detecting cylindrically shaped lipids within mini-microdomains of ~15 nm in length along the perimeter of reconstituted vesicles (Vogtt et al., 2010).

Form reveals function when placed in context with the dynamics of synaptic phospholipase substrate specificities (Figure 4A). Secretary phospholipase A_2_ isoforms (sPLA_2_, Group IIA and possibly Group V) and PlsEtn-selective phospholipase A_2_ (PlsEtn-PLA_2_) are preferentially activated at sites of vesicular fusion (Kolko et al., 2003; Wei et al., 2003; Takamori et al., 2006). Group IIA sPLA_2_, released at the synapse, displays a low affinity for ester-linked acyl-PCs and a high affinity for ether-linked 1-*O*-alkyl-PCs (Pruzanski et al., 1998; Boyanovsky and Webb, 2009). Intracellular calcium-independent PlsEtn-PLA_2_ preferentially hydrolyzes DHA (22:6) from PlsEtns species (Ramadan et al., 2010). Hydrolysis of the outer leaflets of vesicular membranes (by intracellular PlsEtn-PLA_2_) and of pre-synaptic densities (by sPLA_2,_), releasing AA (20:4) from PC(*O*-16:0/20:4) and DHA (22:6) from PE(*P*-16:0/22:6), would indeed generate highly fusogenic wedge-shaped *lyso*-PAFs and *lyso*-plasmalogens without impacting on neighboring diacyl-PCs. The geometries of *lyso*-PAF PC(*O*-16:0/0:0), *lyso*-PlsEtn PE(P-16:0/0:0), and *lyso*-PlsEtn PE(P-18:0/0:0) are predicted to favor the reversal from negative to positive curvature required for the fusion pore formation (Piomelli et al., 2007; Shin et al., 2010) (Figure 4F). These *lyso*-lipids have also been shown to regulate soluble N-ethylmaleimide-sensitive factor attachment receptor (SNARE) protein complexes embedded both in vesicular (v-SNARE) and pre-synaptic target (t-SNARE) membranes. SNARE complexes direct vesicular fusion with activities modulated by their phospholipid binding partners. Association with acyl-linked *lyso*phospholipids promotes the formation of larger of t-/v-SNARE protein complexes slowing down vesicle fusion while association with *lyso*-PlsEtns enhances the formation of smaller complexes speeding up fusion (Glaser and Gross, 1994). Moreover, the free DHA (22:6) and AA (20:4), released from the *sn*-2 position of ether-linked structural membrane lipids by the actions of sPLA_2_ and PlsEtn-PLA_2_, can themselves signal enhanced basal release of noradrenaline, again apparently through converging feedback mechanisms that activate SNAREs (Darios et al., 2010; Geraldine et al., 2010).

### Enhanced hydrolysis of vesicular PAF precursors and membrane plsetns is mechanistically consistent with the hypothesis that increased vesicular release precipitates vesicular depletion over the course of AD

MS profiling of post-mortem AD brain as well as tissue and synaptosomes isolated from transgenic models of AD and AD genetic risk factors provide strong evidence that this pattern is disrupted over the course of AD (Figures 3A–D). Specifically, a depletion in DHA (22:6), an increase in free AA (20:4), and an accumulation of ether-linked fusogenic *lyso*-phospholipids defined by (16:0) carbons at their *sn*-1 position are consistently detected across datasets (Sanchez-Mejia et al., 2008; Ryan et al., 2009; Astarita et al., 2010, 2011). This particular pattern is remarkably similar to the acute changes in free fatty acids and phospholipid profiles observed following toxin-induced synaptic depletion in snake venom poisoning. Certain snake venoms act to stimulate vesicular fusion, partially by enhancing phospholipase-dependent structural membrane lipid hydrolysis, while simultaneously inhibiting the remodeling of the ether and vinyl-ether linked *lyso*-phospholipid metabolites required for the subsequent reconstitution of vesicular membranes. The net effect is vesicular depletion precipitating acute synaptic failure (Valentin and Lambeau, 2000). This phenomenon can also be artificially induced simply by the addition of the *lyso*-phospholipids at concentrations that inhibit their own remodeling (Rigoni et al., 2005; Rossetto et al., 2006). The phospholipid profiles detected in AD datasets mirror this pattern of synaptic poisoning (Figures 3A–D) albeit over a much longer time course. Changes are consistent with the hypothesis that enhanced vesicular release in patients suffering from mild cognitive impairment (MCI), precedes (and likely precipitates) the vesicular depletion seen in moderate to late stage AD (DeKosky et al., 2002; Truchot et al., 2007). Certainly, acute exposure of primary neurons to Aβ oligomers enhances vesicular fusion and increases the rate of neurotransmitter release while chronic exposure depletes neurons of synaptic vesicles and impairs neurotransmission (Dante et al., 2008; Nimmrich and Ebert, 2009; Parodi et al., 2010).

Enhanced hydrolysis of vesicular PAF precursors and membrane PlsEtns may play a determinative role in mediating this transition. In a series of unbiased lipidomic approaches, Aβ oligomers were found to enhance the rate of 1-*O*-alkyl-linked PC leading to an accumulation of AA (20:4) and *lyso*-PAF (*O*-16:0) both *in vitro* and *in vivo* (Sanchez-Mejia et al., 2008; Ryan et al., 2009). The activities of both PlsEtn-PLA_2_ and sPLA_2_ are elevated in AD brain relative to controls, as measured directly in synaptosomes prepared from post-mortem tissue or assayed in cerebrospinal fluid (Chalbot et al., 2009; Farooqui, 2010). Some ESI/MS profiles indicate that PE(P-16:0/18:1), PE(P-18:1/20:4), PE(P-18:0/22:6), and PE(P-18:1/22:6), but not their acyl-linked PE counterparts are selectively reduced in AD brain (Han et al., 2001) (Figure 3A, Table S1). Interestingly, this linkage specificity does not consistently extend to PlsEtns with (16:0) carbons at the *sn*-1 chain and DHA (22:6) or AA (20:4) at the *sn*-2 position (Han et al., 2001). Together, these variations suggest that critical PE(P-16:0/22:6) and PE(P-16:0/20:4) compositions may be maintained for longer periods of time in AD brain possibly by enhancing the compensatory remodeling of other lipid subsets. These individual compensatory responses are suggested by the variations detected between datasets of relative levels of other AA and DHA-containing structural lipids (Figures 3A–D, Tables S1–S4). The reason for the *sn*-1 specificity is unclear although loss of ApoE, a risk factor associated with AD, has been shown to alter the traffic of polyunsaturated lipids from astrocytes to neurons, biasing the composition of long-chain fatty acids in synaptosome PCs toward fully saturated (16:0) species in null-mutant mice (Igbavboa et al., 2002) (Figure 3B, Table S2). Certainly, PC(16:0/16:0) species increase in ApoE-deficient mice (Igbavboa et al., 2002) (Figure 3B, Table S2). Furthermore, the calcium-independent PLA_2_γ (iPLA_2_γ)isoform, capable of hydrolyzing both *sn*-1 and *sn*-2 fatty acid chains of diacyl phospholipids and *sn*-2 chains of *O*-alkylacylphospholipids, shows specificity for (16:0) carbon chains (Yan et al., 2005). iPLA_2_ activity is downregulated in some AD patients and this may also contribute to the enrichment in PC(16:0/16:0), PAF(*O*-16:0/2:0), and *lyso*-PAF(*O*-16:0/0:0) detected in some MS datasets (Talbot et al., 2000; Ryan et al., 2009) (Figure 3B, Table S2). These responses would, in turn, be expected to render new vesicles more fusion-resistant over time in the face of ongoing DHA depletion.

### Reductions in PE(P-16:0/22:6) and PE(P-16:0/20:4) correlate with severity of AD cognitive impairment

Not all profiles, however, detect sparing of PE(*P*-16:0/22:6) and PE(*P*-16:0/20:4) in AD (Figure 3A, Table S1). In studies where these species are reduced, loss correlates with clinical dementia scores but, interestingly, not always with post-mortem AD pathology (Han et al., 2001). Patients with less Aβ and tau pathology but a greater reduction in PlsEtns are more impaired than those with exacerbated primary pathology but little change in PlsEtns profile (Han et al., 2001). Similarly, enhanced turnover of structural PlsEtns and PCs, manifested by an accumulation of smaller *lyso*-plasmalogens and choline-containing metabolites detected by magnetic resonance spectroscopy, correlates with more severe cognitive impairment, deterioration, and psychosis (Sweet et al., 2002). Together, these data point to a primary impact of PlsEtn deficiency on synaptic function and highlight PE(*P*-16:0/22:6) and PE(*P*-16:0/20:4) and to a lesser extent *lyso*-PAF PC(*O*-16:0/0:0) as critical phospholipids species regulating vesicular fusion at the membrane level.

## Pattern 2: breakdown of the second messenger glycerophospholipidome: aberrant phospholipid second messenger signaling cascades can transduce AD pathology and enhance Aβ biogenesis

### Depletions in PI(16:0/20:4), PI(16:0/22:6), and PI(18:0/22:6) likely accelerate Aβ biogenesis

Metabolism of structural membrane PIs and 1-*O*-alkyl PAF precursors generates powerful lipid second messengers. Differential distribution of phosphorylated PI metabolites is required for synergistically assembling and remodeling the SNARE complexes providing a second means by which synaptic phospholipids may directly regulate neurotransmission. PI(4,5)P_2_ and PI(3,4,5)P_3_ are enriched in plasma membrane with 16:0/18:1, 18:0/20:4, and 18:0/22:6 isoforms predominating (Igbavboa et al., 2002). PI(4)P is found more frequently in Golgi membranes while PI(3)P and PI(3,5)P_2_ are enriched in endosomal membranes (Lasiecka et al., 2009). In yeast, PI(3)P both enhances the capacity of membrane-bound SNARES to drive fusion in the absence of SNARE chaperones as well as synergistically activates SNARE chaperones to recruit Vam7p into fusion-competent complexes. PI(3)P has also been shown to be required for the subsequent SNARE complex disassembly once fusion is complete and cargo released (Mima and Wickner, 2009a,b). In neurons, this process further depends upon interaction with PI(4,5)P_2_ found in highest concentrations at pre-synaptic densities. Decreasing PI(4,5)P_2_ abolishes the ability of the v-SNARE-regulator, synaptotagmin-1, to interact with both the pre-synaptic membrane and t-SNARE pre-complexes thereby preventing calcium-dependent release of neurotransmitter from the pre-synaptic active zone (Lee et al., 2010).

Again, these critical compositions are disrupted in AD (Figure 3D, Table S4). Early studies detected a reduction in overall PI content in the temporal cortex of AD patients post-mortem (Stokes and Hawthorne, 1987). LC-ESI-MS analysis of ApoE-deficient mice has identified a key molecular specificity in the depletion of PI(16:0/20:4), PI(16:0/22:6), and PI(18:0/22:6) species at synaptic membranes coupled, in some profiles, with an aberrant increase in PI(18:0/20:4) (Igbavboa et al., 2002; Chan et al., 2012). These changes are not only predicted to impair neurotransmitter release but also to enhance γ-secretase activity, responsible for cleavage of APP to Aβ. PI(3)P(16:0/16:0), PI(4)P(16:0/16:0), PI(3,4)P_2_(16:0/16:0), PI(4,5)P_2_(16:0/16:0), and PI(3,4,5)P_3_(16:0/16:0), but not PI(16:0/16:0), PI(5)P(16:0/16:0), or IP_3_ inhibit γ-secretase activity *in vitro* (Osawa et al., 2008). Although carbon chain specificity has yet to be assessed, it is reasonable to assume that the depletion of PI(16:0/20:4) and PI(16:0/22:6), precipitated by loss of ApoE function, will also contribute to accelerating the processing of synaptotoxic Aβ peptides by γ-secretases.

### Progressive depletion of PI(4,5)P_2_ transiently increases vesicular fusion and chronically impairs neurotransmitter release

Metabolic interdependency is, however, underlined by the fact that the familial AD mutations in presenilin 1 and 2, in turn, impair PI(4,5)P_2_ metabolism effectively reducing levels in vesicular and pre-synaptic membranes (Landman et al., 2006). Moreover, in experimental models of AD, PI(4,5)P_2_ is downregulated in response to synaptotoxic oligomeric Aβ_42_ (but not monomeric or fibrillar configurations). This decrease is mediated, in part, by increases at the pre-synaptic active zone in the Ca^2+^dependent activation of PLC hydrolyzing PI(4,5)P_2_ to DAG and IP_3_ as well as increased activity of synaptojanin 1, the primary PI(4,5)P_2_ phosphatase present at neuronal synapses (Berman et al., 2008). In axons, the hydrolysis of PI(4,5)P_2_ by synaptojanin 1, drives the inner leaflet to adopt a negative curvature that embraces trafficking vesicles during fusion (Cremona et al., 1999). Such activity provides another lipid-centric mechanism by which oligomeric Aβ_42_ acutely increases vesicular fusion then chronically impairs neurotransmitter release (Dante et al., 2008; Nimmrich and Ebert, 2009; Parodi et al., 2010).

### Intraneuronal accumulation of PC(O-16:0/2:0) and AA disrupt tau processing and signal neuronal loss

Aβ_42_ has also been shown to activate cPLA_2_ promoting its calcium-dependent translocation to multiple subcellular membranes (Lee et al., 2011). cPLA_2_ preferentially hydrolyzes AA (20:4) from the *sn*-2 position of 1-*O*-alkyl-2-arachidonoyl- and 1-*O*-acyl-2-arachidonoyl-glycerophospholipids (Kita et al., 2006) (Figure 1D). The alkyl-*lyso*phospholipid backbone can then be modified by LPCAT1 at the plasma membrane to either regenerate structural membrane lipids or produce 1-*O*-acetyl-linked PAF second messengers (Shindou et al., 2007; Harayama et al., 2008) (Figure 1D). LPCAT activity has also been shown to increase in AD (Ross et al., 1998), notably in the posterior-temporal entorhinal cortex, a region characterized by the earliest tau pathology (Bierer et al., 1995). We have shown that in AD temporal cortex, transgenic models of AD, and human neurons, the regeneration of structural membrane lipids from this backbone is impaired (Ryan et al., 2009). In the presence of Aβ_42_, LPCATs appear preferentially to utilize acetyl-CoA over acyl-CoA converting *lyso*-PAFs to PAFs and not back to *O*-alkylacylglycerophosphocholine structural lipids (Ryan et al., 2009). Unbiased neurolipidomic approaches have detected both a net increase in the release of free AA (20:4) and elevations in intraneuronal *lyso*-PAF(*O*-16:0/0:0) and PC(*O*-16:0/2:0) (PAF) in AD patients, two different transgenic models of AD, and neuronal cultures exposed to Aβ_42_ (Kriem et al., 2005; Sanchez-Mejia et al., 2008; Ryan et al., 2009) (Figure 3B, Table S2). Acute intraneuronal accumulation of PC(*O*-16:0/2:0) but not PAF species with other *sn*-1 carbon chains initiates an endoplasmic reticulum stress-dependent signaling cascade culminating in the hyperphosphorylation of tau on Alzheimer Disease-specific epitopes by cyclin-dependent kinase 5 (Ryan et al., 2008, 2009). If concentrations remain elevated, PC(*O*-16:0/2:0) signals a calpain-caspase cascade resulting in neuronal death (Ryan et al., 2007, 2008, 2009). While genetic ablation, knockdown, or pharmacological inhibition of cPLA_2_ activation completely attenuates Aβ_42_ neurotoxicity; blocking the different metabolic arms of the AA (20:4) cascade or preventing the accumulation of PC(*O*-16:0/2:0) PAF confers only partial protection suggesting that both the AA cascade and PC(*O*-16:0/2:0) PAF pathways act synergistically to transduce Aβ_42_ neurotoxicity (Kriem et al., 2005; Firuzi et al., 2008; Sanchez-Mejia et al., 2008; Ryan et al., 2009). Thus, structural membrane metabolism may be more than a biomarker of dementia. Accumulation of specific choline-containing phospholipid metabolites detected *in vivo* by magnetic resonance spectroscopy (Klein, 2000; Sweet et al., 2002) may contribute directly to signaling cognitive decline.

## Pattern 3: the frankensteinian synapse: dendritic deformation and DHA

### Lipid raft movement is regulated by dynamic changes in peri- and post-synaptic phospholipid compositions

**Figure 5:**
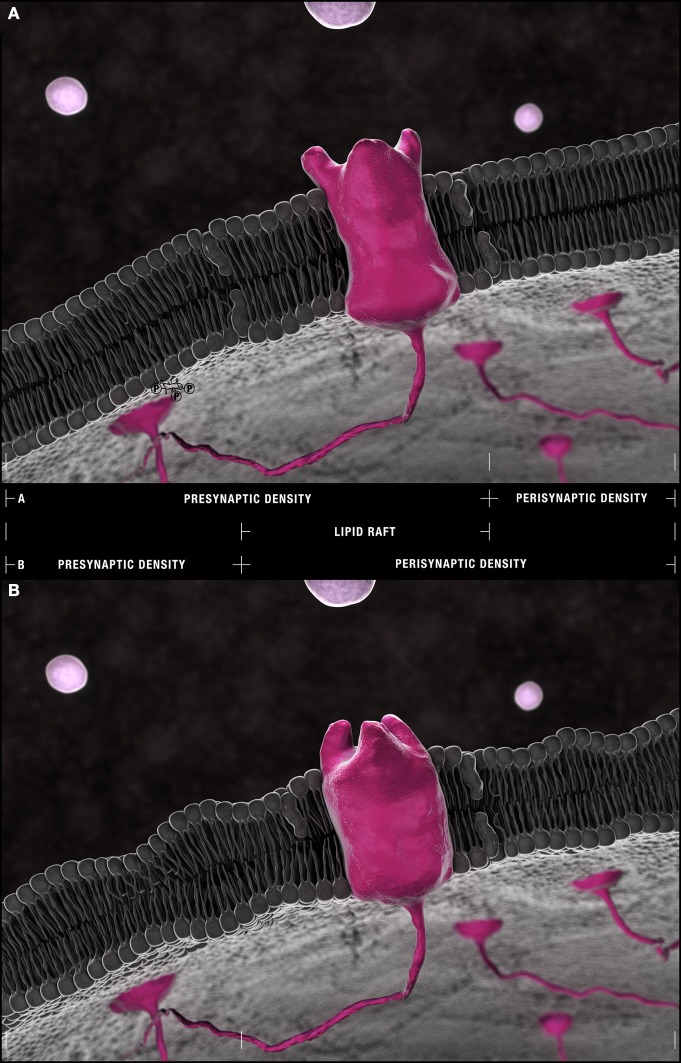
**The Frankensteinian dendritic spine. (A)** Schematic model of a healthy spine. Species within the outer leaflet of post-synaptic densities are more consistently defined by either saturated (16:0) or (18:0) carbon chains at their *sn*-1 and DHA (22:6) at their *sn*-2 positions. PS(18:0/22:6) is more frequently found within the inner leaflet. The lipid raft domains that float freely through these densities and shuttle receptors and their effectors to and from peri- and post-synaptic compartments are depleted of phospholipids relative to non-raft domains yet enriched in cholesterol and sphingolipid isoforms. Their residual phospholipid constituents favor saturated palmitic acid (16:0) and stearic acid (18:0) and mono-unsaturated palmitoleic acid (16:1) and eicosenoic (20:1) fatty acyl chains with PC head-groups predominating. (22:6)–containing PEs and PlsEtns in synaptosomal membranes are found in regions of greatest curvature and highest cholesterol content. This pattern suggests an accumulation of PE(16:0/22:6), PE(18:0/22:6), PlsEtn(16:0/22:6), PlsEtn(18:0/22:6) in the outer leaflet and elastic PS(18:0/22:6) in the inner leaflet of post-synaptic densities. **(B)** The Frankensteinian AD spine. Aberrant changes in lipid composition in the face of ongoing DHA depletion are fundamentally change the post-synaptic profile. The potential impact of these changes is presented in this schematic likely manifesting as a thinner dendritic spine and with bulkier lipid rafts more susceptible to drift from pre-synaptic densities into perisynaptic domains given the (1) loss of PI and PI metabolites from the inner leaflet, (2) progressive reduction in PE(16:0/22:6), PE(18:0/22:6), PlsEtn(16:0/22:6), PlsEtn(18:0/22:6), and PS(18:0/22:6) throughout the bilayer, and (3) aberrant remodeling and accumulation of PS(22:6/22:6) at the outer leaflet demonstrated by neurolipidomic approaches. Within lipid rafts, the further accumulation of saturated (16:0/16:0) side chains in choline-containing lipids at the expense of monounsaturated chains is predicted to alter receptor conformation with the loss of enforced negative curvature by mono and polyunsaturated side chains in direct apposition [compare the configuration of an idealized receptor binding site in **(A)** and **(B)**].

Finally, neurolipidomic profiling combined with high resolution imaging approaches are also revealing a reorganization of post-synaptic microdomains in AD mechanistically associated with the dendritic spine deformation and dysfunction (Tackenberg et al., 2009). Like pre-synaptic and vesicular membranes, dendritic peri- and post-synaptic densities are enriched in PE and PlsEtn isoforms (Figures 4B–F) but, as discussed above, with a functional topography (Han et al., 2001; Igbavboa et al., 2002; Takamori et al., 2006; Ryan et al., 2009; Axelsen and Murphy, 2010; Brand et al., 2010; Eberlin et al., 2010; Lohmann et al., 2010; Sharman et al., 2010) (Figure 3A, Table S1). Application of STED-FCS and single molecule optical tracking approaches to the study of lipid dynamics in living cells further reveals a remarkable territoriality to the free diffusion of structural lipids through cell membranes suggesting that lipid composition may regulate the direction of lipid raft movement between peri- and post-synaptic densities. Phospholipids appear to be limited in their free diffusion within membranes to subdomains of ~20 nm in diameter (Eggeling et al., 2009; Sahl et al., 2010). The speed at which membrane lipids patrol these territories is cholesterol-assisted and backbone-specific. Sphingolipids, for example, freely diffuse traversing a 3 nm membrane radius of their territory within 3 ms in the absence of cholesterol. When cholesterol is present, they become trapped for up to 17 ms in these same regions. PE(16:0/16:0) pays little heed to cholesterol, diffusing at rates of less than 4 ms regardless of the presence or absence of cholesterol (Sahl et al., 2010). Thus, the enrichment of sphingolipids within cholesterol-rich lipid rafts is likely, in part, ensured by their lethargy in the presence of cholesterol while the presence of PEs (and PlsEtns) and alkylacylPCs at the borders of lipid rafts may reflect a functional interaction with cholesterol (Tables S1, S2). Here, isoform specificity comes into play. Converging artificial membrane reconstitution studies using ^2^H NMR, nuclear Overhauser enhancement spectroscopy in ^1^H magic angle spinning NMR, X-ray diffraction, and solid-state ^2^H NMR strongly suggest that cholesterol exhibits an aversion for DHA and the unsaturated 1-*O*-alkyl chains of PAF precursor lipids. Rather, cholesterol favors solvation with saturated (16:0) or (18:0) chains (Brzustowicz et al., 1999, 2002a,b; Shaikh et al., 2002; Pitman et al., 2004; Kusumi et al., 2010). These properties posit a “slip-stream” model of raft movement. Sphingolipid-rich microdomains likely move away from membrane regions where their cholesterol constituents come into apposition with the *sn*-2 DHA (22:6) chains of PE(16:0/22:6), PE(18:0/22:6), PE(P-16:0/22:6), PE(P-18:0/22:6), and PS(18:0/22:6) or the hexadecyl and octadecyl alcohols of 1-*O*-linked PEs and PCs. They likely move toward regions with companionable PC(16:0/18:1), PS(16:0/18:1), and PE(16:0/18:1) (Tables S1–S3). One could also envisage that the radial rotation of PE(16:0/22:6), for example, pirouetting to solvate its 16:0 *sn*-1 hydrocarbon with cholesterol could also create eddies and currents in dendritic microdomains promoting directional raft movement through the bilayer schematically presented at a single time point in Figure 5A.

### Compositional specificity in DHA depletion morphologically deforms dendritic spines

Disruptions in these patterns are associated with both impairment of lateral trafficking of receptors along dendritic membranes and morphological deformation of peri- and post-synaptic densities in AD and animal models of AD (Figure 5B). It is well-established that DHA (22:6) concentrations decrease in AD brain, liver, and AD risk models (Farooqui et al., 2007; Lukiw and Bazan, 2008; Pomponi and Bria, 2008; Pauwels et al., 2009; Astarita et al., 2010; Seshadri et al., 2010). Depletion occurs again with remarkable isoform specificity. For example, in synaptic membranes isolated from ApoE-deficient mice, DHA-containing PS(18:0/22:6), PS(22:5/22:6), PC(18:1/22:6), PI(16:0/22:6), and PI(18:0/22:6) species are reduced yet di-polyunsaturated PS(22:6/22:6), PE(22:6/22:6), and PC(22:6/22:6) species are enhanced relative to wild-types (Igbavboa et al., 2002) (Figures 3A–C, Tables S1–S3). Further, in some MCI patients, most AD patients, and APP/PS-1 double transgenic mice, a reduction in flippase activity, responsible for translocating PS isoforms within synaptic bilayers is reduced resulting in the accumulation of PS at the outer leaflet of synaptosomal membranes (Bader Lange et al., 2008, 2010). Because phospholipid length is directly proportional to the number of carbon atoms yet inversely proportional to the number of double bonds in each carbon chain (Shaikh et al., 2009), the combinations of the regional enrichment of PS(22:6/22:6), PE(22:6/22:6), and PC(22:6/22:6), the depletion of other DHA-containing isoforms throughout post-synaptic membranes, and the reduction of PS(18:0:/22:6) at the inner leaflet would most likely thin regions of peri- and post-synaptic densities while making integral lipid rafts more bulky and less mobile (Figure 5B). These changes are predicted to impair synaptic transmission. This neurolipidomic interpretation inferred from phospholipid composition is consistent with previous hypotheses based on observations of physicochemical lipid properties (Piomelli et al., 2007; Pomponi and Bria, 2008). Certainly transgenic mice ectopically expressing human tau exhibit thinner apical and basal dendritic spines associated with a reduction in synaptic strength (Dickstein et al., 2010). This pattern is also consistent with the morphological deformation, dystrophy, and ultimate loss of dendritic spines detected by Golgi impregnation and electron microscopy of post-mortem AD cortex (Baloyannis et al., 2007).

### Converging metabolic disruptions in PI, PE, plsetns metabolism and DHA bioavailability is predicted to impair receptor trafficking between peri- and post-synaptic densities

DHA (22:6) depletion may also directly impact upon receptor density and chemical reception at post-synaptic densities. Aβ oligomers contribute to the impairment of synaptic transmission, in part, by disrupting the endocytosis and trafficking of AMPA and NMDA receptors at dendritic spines (Durakoglugil et al., 2009; Rui et al., 2010). *In vitro*, receptors are both rapidly removed from post-synaptic densities and fail to re-insert during synaptic potentiation following exposure of hippocampal neurons to oligomeric Aβ (Rui et al., 2010). Although underlying mechanisms have only begun to be elucidated, the reduction in PI metabolites and the depletion of DHA from PE and PlsEtns isoforms detected across multiple neurolipidomic datasets are implicated (Figures 3A,D, Tables S1, S4). In dendrites, PI(3,4,5)P_3_, generated, in large part, through the actions of class I phosphatidylinositol-3-kinases (PI3Ks), is required to maintain AMPA receptors at post-synaptic densities (Arendt et al., 2010). Inhibition of PI(3,4,5)P_3_ synthesis enhances AMPA receptor mobility such that they drift from post- to perisynaptic sites (Arendt et al., 2010). This biology is likely reinforced by the depletion of PI precursors, notably PI(16:0/20:4), PI(18:0/20:4), PI(16:0/22:6), and PI(18:0/22:6) observed in ApoE-deficient and AD transgenic mice (Igbavboa et al., 2002; Chan et al., 2012). Within this Frankensteinian synapse—a term we have coined to describe the result of this catastrophic transformation brought about by aberrant changes in lipid composition—rafts would be no longer “corralled” within post-synaptic densities but rather would be free to diffuse to and from perisynaptic regions (Figure 5B). In support of this hypothesis, post-synaptic density protein 95 (PSD-95), a specialized scaffolding protein that complexes receptors with cytoskeletal elements at the synapse, is reduced at post-synaptic densities when cellular membranes are experimentally depleted of DHA yet enriched following DHA supplementation (Wurtman et al., 2009; Langelier et al., 2010). Further, recent evidence that ongoing systemic DHA depletion (Astarita et al., 2010) is compensated for by increased MUFA biosynthesis (Astarita et al., 2011) suggest that these critical metabolic disruptions are likely mutually negatively reinforcing thereby playing a determinative role in precipitating the “critical change of state” required for AD conversion.

## Conclusions: what do changes in phospholipid composition tell us about AD synaptic dysfunction?

Advances in genomics have identified genetic determinants of neurodegenerative disease. Direct biochemical investigations have elucidated multiple signaling pathways altered by these genetic determinants leading to cognitive deterioration (Kim and Tsai, 2009; Nimmrich and Ebert, 2009). The combination of genomics with proteomics is being used to map the temporal changes in gene and protein expression that occur during transition from pre-symptomatic to symptomatic disease states. We argue that the next major advance in rational therapeutic design will come from tying the dynamics of the susceptible cellular metabolome into these genomic and proteomic maps of disease. The emerging field of neurolipidomics is identifying patterns of membrane disruption predicted to confer AD risk. In this analysis, three overarching metabolic disturbances were detected by *post-hoc* analysis of 29 independent datasets. These impairments include an increase in the hydrolysis of PAF precursors and membrane PlsEtns coupled with a failure to regenerate these relatively rare alkyl-acyl and alkenyl-acyl structural phospholipid compositions. Initially, pathological disruptions appear to affect specific phospholipids defined by carbon chain length, linkages, and degree of unsaturation. For example, decreases in PE(P-16:0/22:6) and PE(P-16:0/20:40 correlate with disease severity. Moreover, accumulation of specific bioactive PAF and PlsEtns metabolites [i.e., PC(*O*-16:0/2:0) and PE(*P*-16:0/0:0)] are implicated in accelerating tau pathology, enhancing vesicular release leading to vesicular depletion, and signaling neuronal loss. Further depletion of PI(16:0/20:4), PI(16:0/22:6), and PI(18:0/22:6) likely accelerates Aβ_42_ biogenesis at the synapse although this hypothesis still requires direct validation. Finally, converging disruptions in PAF precursor and membrane PlsEtn remodeling, PI, notably PI(4,5)P_2,_ and PE metabolism and DHA bioavailability appear to culminate in catastrophic remodeling of the synapse, mechanistically linked to vesicular depletion, impaired receptor trafficking, and morphological dendritic deformation. It will be essential to test whether intervention into one or more of these metabolic pathways can delay conversion from pre-symptomatic to symptomatic AD in the face of ongoing Aβ_42_ and tau pathology. A better understanding of how cellular bioactive lipids alter susceptibility to driving AD pathologies represents a new, potentially transformative, means of identifying and targeting metabolic determinants of Aβ susceptibility and, in the long-term, an exciting means of potentially promoting AD resistance.

### Conflict of interest statement

The authors declare that the research was conducted in the absence of any commercial or financial relationships that could be construed as a potential conflict of interest.

## Footnotes

^1^A number of excellent reviews have been recently published critically assessing these technological milestones in neurolipidomics (Power and Patel, 2004; Han, 2007, 2010; Piomelli et al., 2007; Hou et al., 2008; Astarita et al., 2009; Brown and Murphy, 2009; Lindner and Naim, 2009; Blanksby and Mitchell, 2010; Bou Khalil et al., 2010; Niemoller and Bazan, 2010; Shevchenko and Simons, 2010; Astarita and Piomelli, 2011; Wood, 2012).

^2^These patterns are based on a comparison of the relative abundance within control datasets (wild-type mouse or normal elderly). References and comparative summary of relative phospholipid abundance within these datasets are presented in Tables S1–S5.

^3^We use the LIPID MAPS classification system nomenclature (Fahy et al., 2011). PC(16:0/18:1), for example, defines a lipid species with a phosphocholine polar head group (PC), an ester linkage at the *sn*-1 position, carbon chains at the *sn*-1 and *sn*-2 positions of 16 and 18 carbons, respectively, of which the *sn*-1 chain is fully saturated (referred to as :0), whereas the *sn*−2 has one double bond (referred to as :1). Alkyl (*O*-) and alkenyl (*P*-) linkages are indicated before the appropriate *sn* chain when discussing PlsEtns, PAFs, or PAF precursors.

^4^PE(P-18:0/20:4) or 1-(1Z-octadecenyl)-2-(5Z,8Z,11Z,14Z-eicosatetraenoyl)- *sn*-glycero-3-phosphoethanolamine.

^5^PE(P-16:0/22:6) or 1-(1Z-hexadecenyl)-2-(4Z,7Z,10Z,13Z, 16Z,19Z-docosahexaenoyl)-*sn*-glycero-3-phosphoethanolamine.
