# Supplementary material for: Using neurolipidomics to identify phospholipid mediators of synaptic (dys)function in Alzheimer's Disease
Source: Front Physiol. 2013 Jul 16;4:168. doi: 10.3389/fphys.2013.00168 (PMC3712192; doi:10.3389/fphys.2013.00168)
Supplement: Supplementary file 1 [file Presentation1.PDF]

Using neurolipidomics to identify phospholipid mediators of synaptic (dys)function in Alzheimer's Disease

Steffany A.L. Bennett[1,2,4*], Nicolas Valenzuela[2-4], Hongbin Xu[1,2,4], Bettina Franko[1,2,4], Stephen Fai[3.4,] Daniel Figeys[1,4]

[1]Ottawa Institute of Systems Biology, [2]Neural Regeneration Laboratory, Department of Biochemistry, Microbiology, and Immunology, University of Ottawa, Ottawa, Canada, [3]Carleton Immersive Media Studio, Azrieli School of Architecture and Urbanism, [4]CIHR Training Program in Neurodegenerative Lipidomics, Ottawa, Canada

Correspondence:
Dr Steffany A.L. Bennett
Neural Regeneration Laboratory and Ottawa Institute of Systems Biology,
Department of Biochemistry, Microbiology, and Immunology,
University of Ottawa,
451 Smyth Rd.,
Ottawa, ON, K1H 8M5, Canada
sbennet@uottawa.ca

Supplemental Table 1: Changes in phosphoethanolamine composition in neural tissue, synaptic membranes, and synaptic vesicles of postmortem human AD patients and experimental models of AD and AD risk compared to normal elderly (human) or congenic controls (animals models): A comparison of 14 independent neurolipidomic datasets from eight different laboratories

Phosphoethanolamines

Molecular Species[a]	m/z[b]	Relative abundance[c]	Datasets[g]	Cohort	Source[h]	References
**Total PE (diacylphosphatidyl ethanolamines)**		=	$ApoE^{-/-}$	Mouse	SPM	(Igbavboa et al., 2002)
		= = [d]	ApoE ε2 KI	Mouse	Brain	(Sharman et al., 2010)
		= = [d]	ApoE ε3 KI	Mouse	Brain	(Sharman et al., 2010)
		=↓ [d]	ApoE ε4 KI	Mouse	Brain	(Sharman et al., 2010)
		↓↓[e]	AD	Human	FCx	(Han et al., 2001)
		↓	AD	Human	FCx	(Chan et al., 2012)
		↓↓ [e]	AD	Human	PCx	(Han et al., 2001)
		↓↓ [e]	AD	Human	TCx	(Han et al., 2001)
		=	AD	Human	ECx	(Chan et al., 2012)
		= = [e]	AD	Human	Crb	(Han et al., 2001)
		=	AD	Human	Crb	(Chan et al., 2012)
		= = [f]	APP_{sw}	Mouse	Crb	(Han et al., 2001)
		= ↓ [f]	APP_{sw}	Mouse	Cx	(Han et al., 2001)
		=	APP_{Sw}	Mouse	Brain	(Chan et al., 2012)
		=	PS1	Mouse	Brain	(Chan et al., 2012)
		=	PS1/APP_{Sw}	Mouse	Brain	(Chan et al., 2012)
PE(16:1/0:0)	450	=	PS1	Mouse	Brain	(Chan et al., 2012)
		=	APP_{Sw}	Mouse	Brain	(Chan et al., 2012)
		=	PS1/APP_{Sw}	Mouse	Brain	(Chan et al., 2012)
		=	AD	Human	FCx	(Chan et al., 2012)
		=	AD	Human	ECx	(Chan et al., 2012)
		=	AD	Human	Crb	(Chan et al., 2012)
PE(16:0/0:0)	452	=	PS1	Mouse	Brain	(Chan et al., 2012)
		=	APP_{Sw}	Mouse	Brain	(Chan et al., 2012)
		=	PS1/APP_{Sw}	Mouse	Brain	(Chan et al., 2012)
		=	AD	Human	FCx	(Chan et al., 2012)
		=	AD	Human	ECx	(Chan et al., 2012)
		=	AD	Human	Crb	(Chan et al., 2012)
PE(18:2/0:0)	476	=	PS1	Mouse	Brain	(Chan et al., 2012)
		↓	APP_{Sw}	Mouse	Brain	(Chan et al., 2012)
		↓	PS1/APP_{Sw}	Mouse	Brain	(Chan et al., 2012)
		=	AD	Human	FCx	(Chan et al., 2012)
		=	AD	Human	ECx	(Chan et al., 2012)
		=	AD	Human	Crb	(Chan et al., 2012)
PE(18:1/0:0)	478	=	PS1	Mouse	Brain	(Chan et al., 2012)
		=	APP_{Sw}	Mouse	Brain	(Chan et al., 2012)
		↓	PS1/APP_{Sw}	Mouse	Brain	(Chan et al., 2012)
		=	AD	Human	FCx	(Chan et al., 2012)
		=	AD	Human	ECx	(Chan et al., 2012)
		=	AD	Human	Crb	(Chan et al., 2012)
PE(18:0/0:0)	480	=	PS1	Mouse	Brain	(Chan et al., 2012)
		↓	APP_{Sw}	Mouse	Brain	(Chan et al., 2012)
		=	PS1/APP_{Sw}	Mouse	Brain	(Chan et al., 2012)
		=	AD	Human	FCx	(Chan et al., 2012)
		↓	AD	Human	ECx	(Chan et al., 2012)
		=	AD	Human	Crb	(Chan et al., 2012)
PE(16:1/16:1)	686	=	PS1	Mouse	Brain	(Chan et al., 2012)
		=	APP_{Sw}	Mouse	Brain	(Chan et al., 2012)

		=	PS1/APP_{Sw}	Mouse	Brain	(Chan et al., 2012)
		=	AD	Human	FCx	(Chan et al., 2012)
		=	AD	Human	ECx	(Chan et al., 2012)
		=	AD	Human	Crb	(Chan et al., 2012)
PE(16:0/16:1)	688	=	PS1	Mouse	Brain	(Chan et al., 2012)
		=	APP_{Sw}	Mouse	Brain	(Chan et al., 2012)
		↓	PS1/APP_{Sw}	Mouse	Brain	(Chan et al., 2012)
		↑	AD	Human	FCx	(Chan et al., 2012)
		=	AD	Human	ECx	(Chan et al., 2012)
		=	AD	Human	Crb	(Chan et al., 2012)
PE(16:0/16:0)	690	=	PS1	Mouse	Brain	(Chan et al., 2012)
		=	APP_{Sw}	Mouse	Brain	(Chan et al., 2012)
		↓	PS1/APP_{Sw}	Mouse	Brain	(Chan et al., 2012)
		=	AD	Human	FCx	(Chan et al., 2012)
		=	AD	Human	ECx	(Chan et al., 2012)
		=	AD	Human	Crb	(Chan et al., 2012)
PE(16:1/18:1)	714	=	PS1	Mouse	Brain	(Chan et al., 2012)
		=	APP_{Sw}	Mouse	Brain	(Chan et al., 2012)
		=	PS1/APP_{Sw}	Mouse	Brain	(Chan et al., 2012)
		ND = [e]	AD	Human	FCx	(Han et al., 2001)
		=	AD	Human	FCx	(Chan et al., 2012)
		ND = [e]	AD	Human	PCx	(Han et al., 2001)
		ND = [e]	AD	Human	TCx	(Han et al., 2001)
		=	AD	Human	ECx	(Chan et al., 2012)
		=	AD	Human	Crb	(Chan et al., 2012)
		ND = [e]	AD	Human	Crb	(Han et al., 2001)
PE(16:0/18:1)	716	=	$ApoE^{-/-}$	Mouse	SPM	(Igbavboa et al., 2002)
		= = [e]	AD	Human	FCx	(Han et al., 2001)
		↓	AD	Human	FCx	(Chan et al., 2012)
		= = [e]	AD	Human	PCx	(Han et al., 2001)
		= = [e]	AD	Human	TCx	(Han et al., 2001)
		=	AD	Human	ECx	(Chan et al., 2012)
		= = [e]	AD	Human	Crb	(Han et al., 2001)
		=	AD	Human	Crb	(Chan et al., 2012)
		= = [f]	APP_{sw}	Mouse	Crb	(Han et al., 2001)
		= = [f]	APP_{sw}	Mouse	Cx	(Han et al., 2001)
		↑	PS1	Mouse	Brain	(Chan et al., 2012)
		=	APP_{Sw}	Mouse	Brain	(Chan et al., 2012)
		=	PS1/APP_{Sw}	Mouse	Brain	(Chan et al., 2012)
		CON	Wild-type	Mouse	Hip	(Axelsen and Murphy, 2010)
		CON	Wild-type	Mouse	Cx	(Axelsen and Murphy, 2010)
PE(16:0/18:0)	718	=	PS1	Mouse	Brain	(Chan et al., 2012)
		=	APP_{Sw}	Mouse	Brain	(Chan et al., 2012)
		=	PS1/APP_{Sw}	Mouse	Brain	(Chan et al., 2012)
		=	AD	Human	FCx	(Chan et al., 2012)
		=	AD	Human	ECx	(Chan et al., 2012)
		=	AD	Human	Crb	(Chan et al., 2012)
PE(16:0/20:6)	735	↓	PS1	Mouse	Brain	(Chan et al., 2012)
		=	APP_{Sw}	Mouse	Brain	(Chan et al., 2012)
		↓	PS1/APP_{Sw}	Mouse	Brain	(Chan et al., 2012)
		=	AD	Human	FCx	(Chan et al., 2012)
		=	AD	Human	ECx	(Chan et al., 2012)
		=	AD	Human	Crb	(Chan et al., 2012)
PE(16:0/20:5)	737	=	PS1	Mouse	Brain	(Chan et al., 2012)
		=	APP_{Sw}	Mouse	Brain	(Chan et al., 2012)
		=	PS1/APP_{Sw}	Mouse	Brain	(Chan et al., 2012)
		=	AD	Human	FCx	(Chan et al., 2012)
		↑	AD	Human	ECx	(Chan et al., 2012)
		=	AD	Human	Crb	(Chan et al., 2012)

PE(16:0/20:4)	739	=	$ApoE^{-/-}$	Mouse	SPM	(Igbavboa et al., 2002)
		=ω6	- DHA	Rat	Cx	(Brand et al., 2010)
		= = [e]	AD	Human	FCx	(Han et al., 2001)
		↓	AD	Human	FCx	(Chan et al., 2012)
		= = [e]	AD	Human	PCx	(Han et al., 2001)
		= = [e]	AD	Human	TCx	(Han et al., 2001)
		=	AD	Human	ECx	(Chan et al., 2012)
		= = [e]	AD	Human	Crb	(Han et al., 2001)
		=	AD	Human	Crb	(Chan et al., 2012)
		=	PS1	Mouse	Brain	(Chan et al., 2012)
		=	APP_{Sw}	Mouse	Brain	(Chan et al., 2012)
		=	$PS1/APP_{Sw}$	Mouse	Brain	(Chan et al., 2012)
		= = [f]	APP_{sw}	Mouse	Crb	(Han et al., 2001)
		= = [f]	APP_{sw}	Mouse	Cx	(Han et al., 2001)
PE(18:1/18:2)	741	=↓[e]	AD	Human	FCx	(Han et al., 2001)
		=	AD	Human	FCx	(Chan et al., 2012)
		=↓ [e]	AD	Human	PCx	(Han et al., 2001)
		=↓ [e]	AD	Human	TCx	(Han et al., 2001)
		=	AD	Human	ECx	(Chan et al., 2012)
		= = [e]	AD	Human	Crb	(Han et al., 2001)
		=	AD	Human	Crb	(Chan et al., 2012)
		=	PS1	Mouse	Brain	(Chan et al., 2012)
		=	APP_{Sw}	Mouse	Brain	(Chan et al., 2012)
		=	$PS1/APP_{Sw}$	Mouse	Brain	(Chan et al., 2012)
PE(18:1/18:1) or PE (18:0/18/2)	743	=↓ [e]	AD	Human	FCx	(Han et al., 2001)
		= [k]	AD	Human	FCx	(Chan et al., 2012)
		↓ = [e]	AD	Human	PCx	(Han et al., 2001)
		=↓[e]	AD	Human	TCx	(Han et al., 2001)
		= [k]	AD	Human	ECx	(Chan et al., 2012)
		= [k]	AD	Human	Crb	(Chan et al., 2012)
		= = [e]	AD	Human	Crb	(Han et al., 2001)
		↑ [k]	PS1	Mouse	Brain	(Chan et al., 2012)
		= [k]	APP_{Sw}	Mouse	Brain	(Chan et al., 2012)
		= [k]	$PS1/APP_{Sw}$	Mouse	Brain	(Chan et al., 2012)
		= = [f]	APP_{sw}	Mouse	Crb	(Han et al., 2001)
		= = [f]	APP_{sw}	Mouse	Cx	(Han et al., 2001)
PE(18:0/18:1)	744	=	$ApoE^{-/-}$	Mouse	SPM	(Igbavboa et al., 2002)
		== [e]	AD	Human	FCx	(Han et al., 2001)
		↓	AD	Human	FCx	(Chan et al., 2012)
		= = [e]	AD	Human	PCx	(Han et al., 2001)
		= = [e]	AD	Human	TCx	(Han et al., 2001)
		=	AD	Human	ECx	(Chan et al., 2012)
		↓	AD	Human	Crb	(Chan et al., 2012)
		=↓ [e]	AD	Human	Crb	(Han et al., 2001)
		↑	PS1	Mouse	Brain	(Chan et al., 2012)
		=	APP_{Sw}	Mouse	Brain	(Chan et al., 2012)
		=	$PS1/APP_{Sw}$	Mouse	Brain	(Chan et al., 2012)
		= = [f]	APP_{sw}	Mouse	Crb	(Han et al., 2001)
		= = [f]	APP_{sw}	Mouse	Cx	(Han et al., 2001)
		CON	Wild-type	Mouse	Hip	(Axelsen and Murphy, 2010)
		CON	Wild-type	Mouse	Cx	(Axelsen and Murphy, 2010)
PE(16:1/20:6)	760	=	PS1	Mouse	Brain	(Chan et al., 2012)
		=	APP_{Sw}	Mouse	Brain	(Chan et al., 2012)
		=	$PS1/APP_{Sw}$	Mouse	Brain	(Chan et al., 2012)
		=	AD	Human	FCx	(Chan et al., 2012)
		=	AD	Human	ECx	(Chan et al., 2012)
		=	AD	Human	Crb	(Chan et al., 2012)
PE(16:0/22:6)	763	=	$ApoE^{-/-}$	Mouse	SPM	(Igbavboa et al., 2002)

		CON	Wild-type	Rat	SV	(Takamori et al., 2006)
		↓ω3	- DHA	Rat	Cx	(Brand et al., 2010)
		↓ = [e]	AD	Human	FCx	(Han et al., 2001)
		↓ [l]	AD	Human	FCx	(Chan et al., 2012)
		↓↓ [e]	AD	Human	PCx	(Han et al., 2001)
		↓↓ [e]	AD	Human	TCx	(Han et al., 2001)
		=	AD	Human	ECx	(Chan et al., 2012)
		=	AD	Human	Crb	(Chan et al., 2012)
		= ↓ [e]	AD	Human	Crb	(Han et al., 2001)
		=	PS1	Mouse	Brain	(Chan et al., 2012)
		=	APP_{Sw}	Mouse	Brain	(Chan et al., 2012)
		=	PS1/APP_{Sw}	Mouse	Brain	(Chan et al., 2012)
		= = [f]	APP_{sw}	Mouse	Crb	(Han et al., 2001)
		= ↓ [f]	APP_{sw}	Mouse	Cx	(Han et al., 2001)
PE(16:0/22:5)	765	=	$ApoE^{-/-}$	Mouse	SPM	(Igbavboa et al., 2002)
		↑ω6	- DHA	Rat	Cx	(Brand et al., 2010)
		= = [e]	AD	Human	FCx	(Han et al., 2001)
		↓	AD	Human	FCx	(Chan et al., 2012)
		= = [e]	AD	Human	PCx	(Han et al., 2001)
		= = [e]	AD	Human	TCx	(Han et al., 2001)
		↑	AD	Human	ECx	(Chan et al., 2012)
		=	AD	Human	Crb	(Chan et al., 2012)
		= ↓ [e]	AD	Human	Crb	(Han et al., 2001)
		=	PS1	Mouse	Brain	(Chan et al., 2012)
		=	APP_{Sw}	Mouse	Brain	(Chan et al., 2012)
		=	PS1/APP_{Sw}	Mouse	Brain	(Chan et al., 2012)
		= = [f]	APP_{sw}	Mouse	Crb	(Han et al., 2001)
		= = [f]	APP_{sw}	Mouse	Cx	(Han et al., 2001)
PE(18:0/20:4)<br><br>Note: Species PE(38:4) could equally be PE(16:0/22:4) or PE(18:0/20:4). Both species are distinguished by Igbavboa et al., 2002.	767	=	$ApoE^{-/-}$	Mouse	SPM	(Igbavboa et al., 2002)
		↑ω6	- DHA	Rat	Cx	(Brand et al., 2010)
		CON	Wild-type	Rat	SV	(Takamori et al., 2006)
		↓ = [e]	AD	Human	FCx	(Han et al., 2001)
		↓	AD	Human	FCx	(Chan et al., 2012)
		= = [e]	AD	Human	PCx	(Han et al., 2001)
		= = [e]	AD	Human	TCx	(Han et al., 2001)
		=	AD	Human	ECx	(Chan et al., 2012)
		=	AD	Human	Crb	(Chan et al., 2012)
		= ↓ [e]	AD	Human	Crb	(Han et al., 2001)
		= = [f]	APP_{sw}	Mouse	Crb	(Han et al., 2001)
		=	PS1	Mouse	Brain	(Chan et al., 2012)
		=	APP_{Sw}	Mouse	Brain	(Chan et al., 2012)
		=	PS1/APP_{Sw}	Mouse	Brain	(Chan et al., 2012)
		= = [f]	APP_{sw}	Mouse	Cx	(Han et al., 2001)
		CON	Wild-type	Mouse	Hiip	(Axelsen and Murphy, 2010)
		CON	Wild-type	Mouse	Cx	(Axelsen and Murphy, 2010)
PE(16:0/22:4)	767	=	$ApoE^{-/-}$	Mouse	SPM	(Igbavboa et al., 2002)
PE(18:0/20:3)	769	↓	PS1	Mouse	Brain	(Chan et al., 2012)
		=	APP_{Sw}	Mouse	Brain	(Chan et al., 2012)
		=	PS1/APP_{Sw}	Mouse	Brain	(Chan et al., 2012)
		=	AD	Human	FCx	(Chan et al., 2012)
		=	AD	Human	ECx	(Chan et al., 2012)
		=	AD	Human	Crb	(Chan et al., 2012)
PE(18:1/20:1)	771	= = [f]	APP_{sw}	Mouse	Crb	(Han et al., 2001)
		= = [f]	APP_{sw}	Mouse	Cx	(Han et al., 2001)
		=	PS1	Mouse	Brain	(Chan et al., 2012)
		=	APP_{Sw}	Mouse	Brain	(Chan et al., 2012)
		=	PS1/APP_{Sw}	Mouse	Brain	(Chan et al., 2012)
		=	AD	Human	FCx	(Chan et al., 2012)

		=	AD	Human	ECx	(Chan et al., 2012)
		=	AD	Human	Crb	(Chan et al., 2012)
PE(18:0/20:1)	773	= = [f]	APP_{sw}	Mouse	Crb	(Han et al., 2001)
		= = [f]	APP_{sw}	Mouse	Cx	(Han et al., 2001)
		=	PS1	Mouse	Brain	(Chan et al., 2012)
		=	APP_{Sw}	Mouse	Brain	(Chan et al., 2012)
		=	$PS1/APP_{Sw}$	Mouse	Brain	(Chan et al., 2012)
		=	AD	Human	FCx	(Chan et al., 2012)
		=	AD	Human	ECx	(Chan et al., 2012)
		=	AD	Human	Crb	(Chan et al., 2012)
PE(18:1/22:6)	789	=	$ApoE^{-/-}$	Mouse	SPM	(Igbavboa et al., 2002)
		↓ω3	- DHA	Rat	Cx	(Brand et al., 2010)
		= ↓ [e]	AD	Human	FCx	(Han et al., 2001)
		=	AD	Human	FCx	(Chan et al., 2012)
		= = [e]	AD	Human	PCx	(Han et al., 2001)
		= = [e]	AD	Human	TCx	(Han et al., 2001)
		=	AD	Human	ECx	(Chan et al., 2012)
		=	AD	Human	Crb	(Chan et al., 2012)
		↓ = [e]	AD	Human	Crb	(Han et al., 2001)
		=	APP_{Sw}	Mouse	Brain	(Chan et al., 2012)
		=	$PS1/APP_{Sw}$	Mouse	Brain	(Chan et al., 2012)
		=	PS1	Mouse	Brain	(Chan et al., 2012)
		= = [f]	APP_{sw}	Mouse	Crb	(Han et al., 2001)
		= = [f]	APP_{sw}	Mouse	Cx	(Han et al., 2001)
PE(18:0/22:6)<br><br>Note: Species PE(40:6) could equally be PE(18:0/22:6) or PE(18:1/22:5). Both species are distinguished by Igbavboa et al., 2002.	790	=	$ApoE^{-/-}$	Mouse	SPM	(Igbavboa et al., 2002)
		CON	Wild-type	Rat	SV	(Takamori et al., 2006)
		↓ω3	- DHA	Rat	Cx	(Brand et al., 2010)
		↓ ↓ [e]	AD	Human	FCx	(Han et al., 2001)
		↓	AD	Human	FCx	(Chan et al., 2012)
		= = [e]	AD	Human	PCx	(Han et al., 2001)
		↓ = [e]	AD	Human	TCx	(Han et al., 2001)
		=	AD	Human	ECx	(Chan et al., 2012)
		=	AD	Human	Crb	(Chan et al., 2012)
		↓ = [e]	AD	Human	Crb	(Han et al., 2001)
		=	APP_{Sw}	Mouse	Brain	(Chan et al., 2012)
		=	$PS1/APP_{Sw}$	Mouse	Brain	(Chan et al., 2012)
		=	PS1	Mouse	Brain	(Chan et al., 2012)
		= = [f]	APP_{sw}	Mouse	Crb	(Han et al., 2001)
		= ↓ [f]	APP_{sw}	Mouse	Cx	(Han et al., 2001)
		CON	Wild-type	Mouse	Hip	(Axelsen and Murphy, 2010)
		CON	Wild-type	Mouse	Cx	(Axelsen and Murphy, 2010)
PE(18:1/22:5)	790	=	$ApoE^{-/-}$	Mouse	SPM	(Igbavboa et al., 2002)
PE(18:1/22:4)<br>or PE(18:0/22:5)	792	↑ω6	- DHA	Rat	Cx	(Brand et al., 2010)
		=	PS1	Mouse	Brain	(Chan et al., 2012)
		=	APP_{Sw}	Mouse	Brain	(Chan et al., 2012)
		=	$PS1/APP_{Sw}$	Mouse	Brain	(Chan et al., 2012)
		↓	AD	Human	FCx	(Chan et al., 2012)
		=	AD	Human	ECx	(Chan et al., 2012)
		=	AD	Human	Crb	(Chan et al., 2012)
PE(18:0/22:4)	794	=	$ApoE^{-/-}$	Mouse	SPM	(Igbavboa et al., 2002)
		=	- DHA	Rat	Cx	(Brand et al., 2010)
		= = [e]	AD	Human	FCx	(Han et al., 2001)
		↓	AD	Human	FCx	(Chan et al., 2012)
		= = [e]	AD	Human	PCx	(Han et al., 2001)
		= = [e]	AD	Human	TCx	(Han et al., 2001)
		↓	AD	Human	ECx	(Chan et al., 2012)
		=	AD	Human	Crb	(Chan et al., 2012)
		= = [e]	AD	Human	Crb	(Han et al., 2001)

		= = [f]	APP_{sw}	Mouse	Crb	(Han et al., 2001)
		= = [f]	APP_{sw}	Mouse	Cx	(Han et al., 2001)
		=	APP_{Sw}	Mouse	Brain	(Chan et al., 2012)
		=	$PS1/APP_{Sw}$	Mouse	Brain	(Chan et al., 2012)
		=	PS1	Mouse	Brain	(Chan et al., 2012)
PE(22:0/18:3)	796	=	PS1	Mouse	Brain	(Chan et al., 2012)
		=	APP_{Sw}	Mouse	Brain	(Chan et al., 2012)
		=	$PS1/APP_{Sw}$	Mouse	Brain	(Chan et al., 2012)
		↓	AD	Human	FCx	(Chan et al., 2012)
		=	AD	Human	ECx	(Chan et al., 2012)
		=	AD	Human	Crb	(Chan et al., 2012)
PE(22:0/18:2)	798	=	PS1	Mouse	Brain	(Chan et al., 2012)
		=	APP_{Sw}	Mouse	Brain	(Chan et al., 2012)
		=	$PS1/APP_{Sw}$	Mouse	Brain	(Chan et al., 2012)
		↓	AD	Human	FCx	(Chan et al., 2012)
		=	AD	Human	ECx	(Chan et al., 2012)
		=	AD	Human	Crb	(Chan et al., 2012)
PE(20:0/22:6)	818	=	PS1	Mouse	Brain	(Chan et al., 2012)
		=	APP_{Sw}	Mouse	Brain	(Chan et al., 2012)
		=	$PS1/APP_{Sw}$	Mouse	Brain	(Chan et al., 2012)
		↓	AD	Human	FCx	(Chan et al., 2012)
		=	AD	Human	ECx	(Chan et al., 2012)
		↑	AD	Human	Crb	(Chan et al., 2012)
PE(20:0/22:5)	820	=	PS1	Mouse	Brain	(Chan et al., 2012)
		=	APP_{Sw}	Mouse	Brain	(Chan et al., 2012)
		=	$PS1/APP_{Sw}$	Mouse	Brain	(Chan et al., 2012)
		=	AD	Human	FCx	(Chan et al., 2012)
		=	AD	Human	ECx	(Chan et al., 2012)
		=	AD	Human	Crb	(Chan et al., 2012)
PE(22:6/22:6)	835	↑	$ApoE^{-/-}$	Mouse	SPM	(Igbavboa et al., 2002)

**Total PlsEtn (plasmalogens)**		=	$ApoE^{-/-}$	Mouse	SPM	(Igbavboa et al., 2002)
		↓	$Ctsd^{-/-}$	Mouse	Brain	(Mutka et al., 2010)
		↓↓ [e]	AD	Human	FCx	(Han et al., 2001)
		↓↓ [e]	AD	Human	PCx	(Han et al., 2001)
		↓↓ [e]	AD	Human	TCx	(Han et al., 2001)
		↓= [e]	AD	Human	Crb	(Han et al., 2001)
		= = [f]	APP_{sw}	Mouse	Crb	(Han et al., 2001)
		=↓ [f]	APP_{sw}	Mouse	Cx	(Han et al., 2001)
PE(P-18:0/0:0)	464	=	PS1	Mouse	Brain	(Chan et al., 2012)
		=	APP_{Sw}	Mouse	Brain	(Chan et al., 2012)
		=	$PS1/APP_{Sw}$	Mouse	Brain	(Chan et al., 2012)
		=	AD	Human	FCx	(Chan et al., 2012)
		=	AD	Human	ECx	(Chan et al., 2012)
		=	AD	Human	Crb	(Chan et al., 2012)
PE(P-20:0/0:0)	492	=	PS1	Mouse	Brain	(Chan et al., 2012)
		=	APP_{Sw}	Mouse	Brain	(Chan et al., 2012)
		=	$PS1/APP_{Sw}$	Mouse	Brain	(Chan et al., 2012)
		=	AD	Human	FCx	(Chan et al., 2012)
		=	AD	Human	ECx	(Chan et al., 2012)
		=	AD	Human	Crb	(Chan et al., 2012)
PE(P-16:0/18:2)	698	=	PS1	Mouse	Brain	(Chan et al., 2012)
		=	APP_{Sw}	Mouse	Brain	(Chan et al., 2012)
		↓	$PS1/APP_{Sw}$	Mouse	Brain	(Chan et al., 2012)
		=	AD	Human	FCx	(Chan et al., 2012)
		=	AD	Human	ECx	(Chan et al., 2012)
		=	AD	Human	Crb	(Chan et al., 2012)
PE(P-16:0/18:1)	701	= = [e]	AD	Human	FCx	(Han et al., 2001)

		= = [e]	AD	Human	PCx	(Han et al., 2001)
		=	AD	Human	FCx	(Chan et al., 2012)
		= ↓ [e]	AD	Human	TCx	(Han et al., 2001)
		=	AD	Human	ECx	(Chan et al., 2012)
		=	AD	Human	Crb	(Chan et al., 2012)
		= = [e]	AD	Human	Crb	(Han et al., 2001)
		= = [f]	APP_{sw}	Mouse	Crb	(Han et al., 2001)
		= = [f]	APP_{sw}	Mouse	Cx	(Han et al., 2001)
		=	PS1	Mouse	Brain	(Chan et al., 2012)
		↓	APP_{Sw}	Mouse	Brain	(Chan et al., 2012)
		=	PS1/APP_{Sw}	Mouse	Brain	(Chan et al., 2012)
		↓	$Ctsd^{-/-}$	Mouse	Brain	(Mutka et al., 2010)
PE(P-16:0/18:0)	703	↓	$Ctsd^{-/-}$	Mouse	Brain	(Mutka et al., 2010)
PE(P-16:1/20:4)	721	= = [f]	APP_{sw}	Mouse	Crb	(Han et al., 2001)
		= = [f]	APP_{sw}	Mouse	Cx	(Han et al., 2001)
PE(P-16:0/20:4)	722	=	$ApoE^{-/-}$	Mouse	SPM	(Igbavboa et al., 2002)
		↑ω6	- DHA	Rat	Cx	(Brand et al., 2010)
		=	AD	Human	FCx	(Chan et al., 2012)
		↓↓ [e]	AD	Human	FCx	(Han et al., 2001)
		=	AD	Human	ECx	(Chan et al., 2012)
		=	AD	Human	Crb	(Chan et al., 2012)
		↓↓ [e]	AD	Human	PCx	(Han et al., 2001)
		↓↓ [e]	AD	Human	TCx	(Han et al., 2001)
		↓ = [e]	AD	Human	Crb	(Han et al., 2001)
		= = [f]	APP_{sw}	Mouse	Crb	(Han et al., 2001)
		= = [f]	APP_{sw}	Mouse	Cx	(Han et al., 2001)
		=	PS1	Mouse	Brain	(Chan et al., 2012)
		=	APP_{Sw}	Mouse	Brain	(Chan et al., 2012)
		=	PS1/APP_{Sw}	Mouse	Brain	(Chan et al., 2012)
		=	$Ctsd^{-/-}$	Mouse	Brain	(Mutka et al., 2010)
		CON	Wild-type	Mouse	Hip	(Axelsen and Murphy, 2010)
		CON	Wild-type	Mouse	Cx	(Axelsen and Murphy, 2010)
PE(P-18:1/18:2)	725	=	PS1	Mouse	Brain	(Chan et al., 2012)
		=	APP_{Sw}	Mouse	Brain	(Chan et al., 2012)
		=	PS1/APP_{Sw}	Mouse	Brain	(Chan et al., 2012)
		↓	$Ctsd^{-/-}$	Mouse	Brain	(Mutka et al., 2010)
		=	AD	Human	FCx	(Chan et al., 2012)
		=	AD	Human	ECx	(Chan et al., 2012)
		=	AD	Human	Crb	(Chan et al., 2012)
PE(P-18:1/18:1) or PE(18:0/18:2)	727	↓↓ [e]	AD	Human	FCx	(Han et al., 2001)
		↑	AD	Human	FCx	(Chan et al., 2012)
		↓↓ [e]	AD	Human	PCx	(Han et al., 2001)
		=	AD	Human	ECx	(Chan et al., 2012)
		=	AD	Human	Crb	(Chan et al., 2012)
		↓↓ [e]	AD	Human	TCx	(Han et al., 2001)
		= ↓ [e]	AD	Human	Crb	(Han et al., 2001)
		↓	$Ctsd^{-/-}$	Mouse	Brain	(Mutka et al., 2010)
		=	PS1	Mouse	Brain	(Chan et al., 2012)
		=	APP_{Sw}	Mouse	Brain	(Chan et al., 2012)
		=	PS1/APP_{Sw}	Mouse	Brain	(Chan et al., 2012)
		= = [f]	APP_{sw}	Mouse	Crb	(Han et al., 2001)
		= = [f]	APP_{sw}	Mouse	Cx	(Han et al., 2001)
PE(P-18:0/18:1) or PE(P-16:0/20:1)	729	= ↓ [e]	AD	Human	FCx	(Han et al., 2001)
		=	AD	Human	FCx	(Chan et al., 2012)
		= ↓ [e]	AD	Human	PCx	(Han et al., 2001)
		= ↓ [e]	AD	Human	TCx	(Han et al., 2001)
		=	AD	Human	ECx	(Chan et al., 2012)
		=	AD	Human	Crb	(Chan et al., 2012)

		=↓ [e]	AD	Human	Crb	(Han et al., 2001)
		↓	Ctsd$^{-/-}$	Mouse	Brain	(Mutka et al., 2010)
		=	PS1	Mouse	Brain	(Chan et al., 2012)
		=	APP_{Sw}	Mouse	Brain	(Chan et al., 2012)
		↓	PS1/APP_{Sw}	Mouse	Brain	(Chan et al., 2012)
		= = [f]	APP_{sw}	Mouse	Crb	(Han et al., 2001)
		= = [f]	APP_{sw}	Mouse	Cx	(Han et al., 2001)
PE(P-18:0/18:0)	730	=	PS1	Mouse	Brain	(Chan et al., 2012)
		=	APP_{Sw}	Mouse	Brain	(Chan et al., 2012)
		↓	PS1/APP_{Sw}	Mouse	Brain	(Chan et al., 2012)
		=	Ctsd$^{-/-}$	Mouse	Brain	(Mutka et al., 2010)
		=	AD	Human	FCx	(Chan et al., 2012)
		=	AD	Human	ECx	(Chan et al., 2012)
		=	AD	Human	Crb	(Chan et al., 2012)
PE(P-16:0/22:6)	747	=	ApoE$^{-/-}$	Mouse	SPM	(Igbavboa et al., 2002)
		↑	Ctsd$^{-/-}$	Mouse	Brain	(Mutka et al., 2010)
		↓ ω3 [f]	- DHA	Rat	Cx	(Brand et al., 2010)
		CON	Wild-type	Rat	SV	(Takamori et al., 2006)
		↓↓ [e]	AD	Human	FCx	(Han et al., 2001)
		↓	AD	Human	FCx	(Chan et al., 2012)
		= = [e]	AD	Human	PCx	(Han et al., 2001)
		↓ = [e]	AD	Human	TCx	(Han et al., 2001)
		=	AD	Human	ECx	(Chan et al., 2012)
		=	AD	Human	Crb	(Chan et al., 2012)
		= = [e]	AD	Human	Crb	(Han et al., 2001)
		=	PS1	Mouse	Brain	(Chan et al., 2012)
		=	PS1/APP_{Sw}	Mouse	Brain	(Chan et al., 2012)
		=	APP_{Sw}	Mouse	Brain	(Chan et al., 2012)
		= = [f]	APP_{sw}	Mouse	Crb	(Han et al., 2001)
		= = [f]	APP_{sw}	Mouse	Cx	(Han et al., 2001)
PE(P-18:1/20:4)	749	=	ApoE$^{-/-}$	Mouse	SPM	(Igbavboa et al., 2002)
		↑ ω6	- DHA	Rat	Cx	(Brand et al., 2010)
		↑	Ctsd$^{-/-}$	Mouse	Brain	(Mutka et al., 2010)
		↓↓ [e,]	AD	Human	FCx	(Han et al., 2001)
		= = [e]	AD	Human	PCx	(Han et al., 2001)
		=	AD	Human	FCx	(Chan et al., 2012)
		↓ = [e]	AD	Human	TCx	(Han et al., 2001)
		=	AD	Human	ECx	(Chan et al., 2012)
		=	AD	Human	Crb	(Chan et al., 2012)
		= = [e]	AD	Human	Crb	(Han et al., 2001)
		=	PS1	Mouse	Brain	(Chan et al., 2012)
		=	PS1/APP_{Sw}	Mouse	Brain	(Chan et al., 2012)
		=	APP_{Sw}	Mouse	Brain	(Chan et al., 2012)
		= = [f]	APP_{sw}	Mouse	Crb	(Han et al., 2001)
		= = [f]	APP_{sw}	Mouse	Cx	(Han et al., 2001)
PE(P-16:0/22:4)<br><br>Note: Species PE(38:4p) could equally be PE(P-16:0/22:4) or PE(P-18:0/20:4).	751	=	ApoE$^{-/-}$	Mouse	SPM	(Igbavboa et al., 2002)
		↑ ω6	- DHA	Rat	Cx	(Brand et al., 2010)
		CON	Wild-type	Rat	SV	(Takamori et al., 2006)
		CON	Wild-type	Mouse	Hip	(Axelsen and Murphy, 2010)
		CON	Wild-type	Mouse	Cx	(Axelsen and Murphy, 2010)
		↓↓ [e]	AD	Human	FCx	(Han et al., 2001)
		=	AD	Human	FCx	(Chan et al., 2012)
		↓ ↓ [e]	AD	Human	PCx	(Han et al., 2001)
		↓ ↓ [e]	AD	Human	TCx	(Han et al., 2001)
		=	AD	Human	ECx	(Chan et al., 2012)
		=	AD	Human	Crb	(Chan et al., 2012)
		=↓ [e]	AD	Human	Crb	(Han et al., 2001)
		= = [f]	APP_{sw}	Mouse	Crb	(Han et al., 2001)

		=	PS1	Mouse	Brain	(Chan et al., 2012)
		=	PS1/APP_{Sw}	Mouse	Brain	(Chan et al., 2012)
		=	APP_{Sw}	Mouse	Brain	(Chan et al., 2012)
		= ↓ [f]	APP_{sw}	Mouse	Cx	(Han et al., 2001)
PE(P-18:0/20:4)	751	↑	$Ctsd^{-/-}$	Mouse	Brain	(Mutka et al., 2010)
PE(P-18:0/20:3)	753	=	PS1	Mouse	Brain	(Chan et al., 2012)
		=	APP_{Sw}	Mouse	Brain	(Chan et al., 2012)
		=	PS1/APP_{Sw}	Mouse	Brain	(Chan et al., 2012)
		=	$Ctsd^{-/-}$	Mouse	Brain	(Mutka et al., 2010)
		=	AD	Human	FCx	(Chan et al., 2012)
		=	AD	Human	ECx	(Chan et al., 2012)
		=	AD	Human	Crb	(Chan et al., 2012)
PE(P-18:1/20:1)	755	=↓ [e]	AD	Human	FCx	(Han et al., 2001)
		=	AD	Human	FCx	(Chan et al., 2012)
		↓↓ [e]	AD	Human	PCx	(Han et al., 2001)
		=↓ [e]	AD	Human	TCx	(Han et al., 2001)
		=	AD	Human	ECx	(Chan et al., 2012)
		=	AD	Human	Crb	(Chan et al., 2012)
		= ↓ [e]	AD	Human	Crb	(Han et al., 2001)
		=	PS1	Mouse	Brain	(Chan et al., 2012)
		=	APP_{Sw}	Mouse	Brain	(Chan et al., 2012)
		=	PS1/APP_{Sw}	Mouse	Brain	(Chan et al., 2012)
		=	$Ctsd^{-/-}$	Mouse	Brain	(Mutka et al., 2010)
		= = [f]	APP_{sw}	Mouse	Crb	(Han et al., 2001)
		= = [f]	APP_{sw}	Mouse	Cx	(Han et al., 2001)
PE(P-18:0/20:1)	757	= = [f]	APP_{sw}	Mouse	Crb	(Han et al., 2001)
		= = [f]	APP_{sw}	Mouse	Cx	(Han et al., 2001)
		=	$Ctsd^{-/-}$	Mouse	Brain	(Mutka et al., 2010)
PE(P-18:1/22:6)	773	=	$ApoE^{-/-}$	Mouse	SPM	(Igbavboa et al., 2002)
		=	$Ctsd^{-/-}$	Mouse	Brain	(Mutka et al., 2010)
		↓ω3	- DHA	Rat	Cx	(Brand et al., 2010)
		↓↓ [e]	AD	Human	FCx	(Han et al., 2001)
		↓ = [e]	AD	Human	PCx	(Han et al., 2001)
		=↓ [e]	AD	Human	TCx	(Han et al., 2001)
		= ↓ [e]	AD	Human	Crb	(Han et al., 2001)
PE(P-18:0/22:6)	775	=	$ApoE^{-/-}$	Mouse	SPM	(Igbavboa et al., 2002)
		↑	$Ctsd^{-/-}$	Mouse	Brain	(Mutka et al., 2010)
		↓ω3	- DHA	Rat	Cx	(Brand et al., 2010)
		↓↓ [e]	AD	Human	FCx	(Han et al., 2001)
		=	AD	Human	FCx	(Chan et al., 2012)
		= = [e]	AD	Human	PCx	(Han et al., 2001)
		↓ = [e]	AD	Human	TCx	(Han et al., 2001)
		=	AD	Human	ECx	(Chan et al., 2012)
		=	AD	Human	Crb	(Chan et al., 2012)
		↓↓ [e]	AD	Human	Crb	(Han et al., 2001)
		=	PS1	Mouse	Brain	(Chan et al., 2012)
		=	APP_{Sw}	Mouse	Brain	(Chan et al., 2012)
		=	PS1/APP_{Sw}	Mouse	Brain	(Chan et al., 2012)
		= = [f]	APP_{sw}	Mouse	Crb	(Han et al., 2001)
		= ↓ [f]	APP_{sw}	Mouse	Cx	(Han et al., 2001)
PE(P-18:0/22:5)	777	↑ω6	- DHA	Rat	Cx	(Brand et al., 2010)
		↑	$Ctsd^{-/-}$	Mouse	Brain	(Mutka et al., 2010)
		↓↓ [e]	AD	Human	FCx	(Han et al., 2001)
		=	AD	Human	FCx	(Chan et al., 2012)
		↓ ↓ [e]	AD	Human	PCx	(Han et al., 2001)
		↓↓ [e]	AD	Human	TCx	(Han et al., 2001)
		=	AD	Human	ECx	(Chan et al., 2012)
		=	AD	Human	Crb	(Chan et al., 2012)

		↓↓ [e]	AD	Human	Crb	(Han et al., 2001)
		=	PS1	Mouse	Brain	(Chan et al., 2012)
		=	APP_{Sw}	Mouse	Brain	(Chan et al., 2012)
		=	$PS1/APP_{Sw}$	Mouse	Brain	(Chan et al., 2012)
		= = [f]	APP_{sw}	Mouse	Crb	(Han et al., 2001)
		= = [f]	APP_{sw}	Mouse	Cx	(Han et al., 2001)
PE(P-18:0/22:4)	779	↓↓ [e]	AD	Human	FCx	(Han et al., 2001)
		=	AD	Human	FCx	(Chan et al., 2012)
		=↓ [e]	AD	Human	PCx	(Han et al., 2001)
		↓↓ [e]	AD	Human	TCx	(Han et al., 2001)
		=	AD	Human	ECx	(Chan et al., 2012)
		=	AD	Human	Crb	(Chan et al., 2012)
		=	$Ctsd^{-/-}$	Mouse	Brain	(Mutka et al., 2010)
		=	PS1	Mouse	Brain	(Chan et al., 2012)
		=	APP_{Sw}	Mouse	Brain	(Chan et al., 2012)
		=	$PS1/APP_{Sw}$	Mouse	Brain	(Chan et al., 2012)
		= = [f]	APP_{sw}	Mouse	Crb	(Han et al., 2001)
		= = [f]	APP_{sw}	Mouse	Cx	(Han et al., 2001)
PE(P-20:4/22:4)	798	=	PS1	Mouse	Brain	(Chan et al., 2012)
		=	APP_{Sw}	Mouse	Brain	(Chan et al., 2012)
		=	$PS1/APP_{Sw}$	Mouse	Brain	(Chan et al., 2012)
		=	AD	Human	FCx	(Chan et al., 2012)
		=	AD	Human	ECx	(Chan et al., 2012)
		=	AD	Human	Crb	(Chan et al., 2012)
PE(P-20:3/22:4)	800	=	PS1	Mouse	Brain	(Chan et al., 2012)
		=	APP_{Sw}	Mouse	Brain	(Chan et al., 2012)
		=	$PS1/APP_{Sw}$	Mouse	Brain	(Chan et al., 2012)
		=	AD	Human	FCx	(Chan et al., 2012)
		=	AD	Human	ECx	(Chan et al., 2012)
		=	AD	Human	Crb	(Chan et al., 2012)
PE(P-20:0/22:6)	802	=	PS1	Mouse	Brain	(Chan et al., 2012)
		↓	APP_{Sw}	Mouse	Brain	(Chan et al., 2012)
		↓	$PS1/APP_{Sw}$	Mouse	Brain	(Chan et al., 2012)
		=	AD	Human	FCx	(Chan et al., 2012)
		=	AD	Human	ECx	(Chan et al., 2012)
		=	AD	Human	Crb	(Chan et al., 2012)
**Total PE(*O*-linked) (alkylacyl-phosphoethanolamines)**		=	$ApoE^{-/-}$	Mouse	SPM	(Igbavboa et al., 2002)
PE(O-16:0/20:4)	725	=	$ApoE^{-/-}$	Mouse	SPM	(Igbavboa et al., 2002)
PE(O-16:0/22:6)	749	=	$ApoE^{-/-}$	Mouse	SPM	(Igbavboa et al., 2002)
PE(O-16:0/22:5)	751	↓	$ApoE^{-/-}$	Mouse	SPM	(Igbavboa et al., 2002)
PE(O-18:0/20:4)	753	=	$ApoE^{-/-}$	Mouse	SPM	(Igbavboa et al., 2002)
PE(O-18:0/22:6)	777	=	$ApoE^{-/-}$	Mouse	SPM	(Igbavboa et al., 2002)
PE(O-18:0/22:5)	779	=	$ApoE^{-/-}$	Mouse	SPM	(Igbavboa et al., 2002)

[a] Stereospecificity of *sn*-1 and *sn*-2 chains was assigned by the authors based on (1) the reported total carbon number and total degree of unsaturation provided in the original datasets (i.e., (Takamori et al., 2006; Chan et al., 2012)) and (2) the most likely isobaric species present in neural cells and brain tissue established (a) empirically in the datasets using standard addition or analysis of *lyso*-form fragment ions attributed to the neutral loss of fatty acyl moieties using MS^2 or MS^3 spectra or (b) predicted in published literature (i.e., (Igbavboa et al., 2002; Whitehead et al., 2007; Smith et al., 2008; Ryan et al., 2009; Hou et al., 2011). In cases where predominant species have yet to be identified empirically or where multiple isobaric species are known to be present in neural membranes, all possible choices are indicated curating for chain length and degree of saturation and stereospecificity considered most likely to appear in mammalian cellular membranes based on prevalence (Miyazaki and Ntambi, 2008) and as predicted using VaLID v1.0.1 (Blanchard et al., 2013).

[b] m/z is reported for [M-H]+ ions (phosphocholines) or [M-H]- ions (all others).

[c] =, ↓,↑ indicate comparisons relative to appropriate controls. CON indicates control data only (i.e., identified in control tissue but not compared to another condition). ND indicates not detected.

[d] First value summarizes changes at 2 months; second value changes at 12 months.

[e] First value summarizes changes in grey matter, second value changes in white matter (of the same patient).

[f] First value summarizes changes at 9 months, second value changes at 18 months.

[g] Datasets:

(1) Profile of isolated rat synaptic vesicles (Takamori et al., 2006);

(2) Profile WT rat hippocampus (Axelsen and Murphy, 2010);

(3) Profile of WT mouse cortex (Axelsen and Murphy, 2010);

(4) - DHA Depletion: The effects of embryonic or postnatal dietary depletion of the DHA precursor α-linolenic acid (18:3n-3) was assessed at postnatal day 1 (neonates) and 1 month old (postnatal) Wistar rats compared to controls fed an adequate diet (Brand et al., 2010);

(5) Comparison of apoliprotein E null mutants ($ApoE^{-/-}$) with wild-type (WT) C57BL/6J mice, 2-3 months of age (Igbavboa et al., 2002);

(6-10) Comparison of APP_{sw} mice transgenic for human APP with the Swedish double mutation K670N/M671L aged between 9-11.5 months of age (onset) with WT mice (Chan et al., 2012); Comparison of this same mouse model at 9 months (onset) and 18 months (symptomatic) of age (Han et al., 2001);

(11) Comparison of mice transgenic for PS1 aged between 9-11.5 months (symptomatic) with WT mice (Chan et al., 2012);

(12) Comparison of double transgenic mice APP_{sw} x PS1 aged between 9-11.5 months (symptomatic) with WT mice (Chan et al., 2012);

(13-23) Comparison of Alzheimer Disease patient with age- and gender-matched controls postmortem in various brain regions (Han et al., 2001; Ryan et al., 2009; Chan et al., 2012);

(24) Comparison of $Ctsd^{-/-}$ cathepsin D null mutant mice with WT controls (Mutka et al., 2010);

(25) Comparison of ApoE ε2, ApoE ε3, and ApoE ε4 knockin of human ApoE variants into the murine ApoE locus (humanized mouse model) with congenic N8 C57Bl/6 x 129P2 mice at 2 (young) and 12 (middle-aged) months of age (Sharman et al., 2010);

(26) Profile of rat Medulla (Lohmann et al., 2010);

(27-28) Comparison of TgCRND8 mice transgenic for human APP with both Swedish (K670N/M671L) and Indiana mutations (V717F) with congenic N4 C57Bl/6 x C3H littermates at both 2 (pre-symptomatic) and 4 (onset) months of age;

(29) Profile of WT mouse cerebrum/brain (Eberlin et al., 2010);

[h] Tissue: Brain, Cerebrum; Cx, Cortex; Crb, cerebellum; ECx, Entorhinal Cortex; FCx, Frontal Cortex/Prefrontal Cortex; Hip, Hippocampus; PCx, Parietal Cortex; SPM, Synaptosomal membranes; SV, Synaptic Vesicle.

Supplemental Table 2: Changes in phosphocholine composition in neural tissue, synaptic membranes, and synaptic vesicles of postmortem human AD patients and experimental models of AD and AD risk compared to normal elderly (human) or congenic controls (animals models): A comparison of 14 independent neurolipidomic datasets from seven different laboratories

Phosphocholines

**Molecular Species**[a]	**m/z**[b]	**Relative abundance**[c]	**Datasets**[g]	**Cohort**	**Source**[h]	**References**
**Total PC (diacyl phosphatidylcholines)**		=	$ApoE^{-/-}$	Mouse	SPM	(Igbavboa et al., 2002)
		= = [d]	ApoE ε2 KI	Mouse	Brain	(Sharman et al., 2010)
		= = [d]	ApoE ε3 KI	Mouse	Brain	(Sharman et al., 2010)
		= = [d]	ApoE ε4 KI	Mouse	Brain	(Sharman et al., 2010)
		=	PS1	Mouse	Brain	(Chan et al., 2012)
		↑	APP_{Sw}	Mouse	Brain	(Chan et al., 2012)
		=	PS1/APP_{Sw}	Mouse	Brain	(Chan et al., 2012)
		=	AD	Human	FCx	(Chan et al., 2012)
		=	AD	Human	ECx	(Chan et al., 2012)
		=	AD	Human	Crb	(Chan et al., 2012)
PC(16:1/0:0)	494	=	PS1	Mouse	Brain	(Chan et al., 2012)
		=	APP_{Sw}	Mouse	Brain	(Chan et al., 2012)
		=	PS1/APP_{Sw}	Mouse	Brain	(Chan et al., 2012)
		=	AD	Human	FCx	(Chan et al., 2012)
		=	AD	Human	ECx	(Chan et al., 2012)
		=	AD	Human	Crb	(Chan et al., 2012)
PC(16:0/0:0)	496	↓	PS1	Mouse	Brain	(Chan et al., 2012)
		=	APP_{Sw}	Mouse	Brain	(Chan et al., 2012)
		=	PS1/APP_{Sw}	Mouse	Brain	(Chan et al., 2012)
		=	AD	Human	FCx	(Chan et al., 2012)
		=	AD	Human	ECx	(Chan et al., 2012)
		=	AD	Human	Crb	(Chan et al., 2012)
PC(18:2/0:0)	520	=	PS1	Mouse	Brain	(Chan et al., 2012)
		=	APP_{Sw}	Mouse	Brain	(Chan et al., 2012)
		=	PS1/APP_{Sw}	Mouse	Brain	(Chan et al., 2012)
		=	AD	Human	FCx	(Chan et al., 2012)
		=	AD	Human	ECx	(Chan et al., 2012)
		=	AD	Human	Crb	(Chan et al., 2012)
PC(18:1/0:0)	522	=	PS1	Mouse	Brain	(Chan et al., 2012)
		=	APP_{Sw}	Mouse	Brain	(Chan et al., 2012)
		↑	PS1/APP_{Sw}	Mouse	Brain	(Chan et al., 2012)
		↓	AD	Human	FCx	(Chan et al., 2012)
		=	AD	Human	ECx	(Chan et al., 2012)
		=	AD	Human	Crb	(Chan et al., 2012)
PC(18:0/0:0)	524	=	PS1	Mouse	Brain	(Chan et al., 2012)
		=	APP_{Sw}	Mouse	Brain	(Chan et al., 2012)
		↑	PS1/APP_{Sw}	Mouse	Brain	(Chan et al., 2012)
		↓	AD	Human	FCx	(Chan et al., 2012)
		=	AD	Human	ECx	(Chan et al., 2012)
		=	AD	Human	Crb	(Chan et al., 2012)
PC(14:0/14:0)	678	CON	Wild-type	Rat	Med	(Lohmann et al., 2010)
PC(14:0/16:0)	706	CON	Wild-type	Rat	Med	(Lohmann et al., 2010)
PC(16:1/16:1)	730	=	PS1	Mouse	Brain	(Chan et al., 2012)
		=	APP_{Sw}	Mouse	Brain	(Chan et al., 2012)
		=	PS1/APP_{Sw}	Mouse	Brain	(Chan et al., 2012)
		=	AD	Human	FCx	(Chan et al., 2012)
		=	AD	Human	ECx	(Chan et al., 2012)
		=	AD	Human	Crb	(Chan et al., 2012)

PC(14:0/18:1)	732	CON	Wild-type	Rat	Med	(Lohmann et al., 2010)
		=	PS1	Mouse	Brain	(Chan et al., 2012)
		↑	APP_{Sw}	Mouse	Brain	(Chan et al., 2012)
		↑	$PS1/APP_{Sw}$	Mouse	Brain	(Chan et al., 2012)
		=	AD	Human	FCx	(Chan et al., 2012)
		=	AD	Human	ECx	(Chan et al., 2012)
		=	AD	Human	Crb	(Chan et al., 2012)
PC(16:0/16:0)	734	↑	$ApoE^{-/-}$	Mouse	SPM	(Igbavboa et al., 2002)
		=	PS1	Mouse	Brain	(Chan et al., 2012)
		=	APP_{Sw}	Mouse	Brain	(Chan et al., 2012)
		=	$PS1/APP_{Sw}$	Mouse	Brain	(Chan et al., 2012)
		=	AD	Human	FCx	(Chan et al., 2012)
		=	AD	Human	ECx	(Chan et al., 2012)
		=	AD	Human	Crb	(Chan et al., 2012)
		CON	Wild-type	Mouse	Hip	(Axelsen and Murphy, 2010)
		CON	Wild-type	Mouse	Cx	(Axelsen and Murphy, 2010)
		CON	Wild-type	Rat	SV	(Takamori et al., 2006)
		CON	Wild-type	Rat	Med	(Lohmann et al., 2010)
PC(16:1/18:2)	756	=	PS1	Mouse	Brain	(Chan et al., 2012)
		↑	APP_{Sw}	Mouse	Brain	(Chan et al., 2012)
		=	$PS1/APP_{Sw}$	Mouse	Brain	(Chan et al., 2012)
		=	AD	Human	FCx	(Chan et al., 2012)
		=	AD	Human	ECx	(Chan et al., 2012)
		=	AD	Human	Crb	(Chan et al., 2012)
PC(16:1/18:2)	758	=	PS1	Mouse	Brain	(Chan et al., 2012)
		=	APP_{Sw}	Mouse	Brain	(Chan et al., 2012)
		=	$PS1/APP_{Sw}$	Mouse	Brain	(Chan et al., 2012)
		=	AD	Human	FCx	(Chan et al., 2012)
		=	AD	Human	ECx	(Chan et al., 2012)
		=	AD	Human	Crb	(Chan et al., 2012)
PC(16:0/18:1)	760	=	$ApoE^{-/-}$	Mouse	SPM	(Igbavboa et al., 2002)
		=	PS1	Mouse	Brain	(Chan et al., 2012)
		↑	APP_{Sw}	Mouse	Brain	(Chan et al., 2012)
		↑	$PS1/APP_{Sw}$	Mouse	Brain	(Chan et al., 2012)
		=	AD	Human	FCx	(Chan et al., 2012)
		=	AD	Human	ECx	(Chan et al., 2012)
		=	AD	Human	Crb	(Chan et al., 2012)
		CON	Wild-type	Rat	SV	(Takamori et al., 2006)
		CON	Wild-type	Rat	Med	(Lohmann et al., 2010)
		CON	Wild-type	Mouse	Hip	(Axelsen and Murphy, 2010)
		CON	Wild-type	Mouse	Cx	(Axelsen and Murphy, 2010)
PC(16:0/18:0)	762	CON	Wild-type	Rat	SV	(Takamori et al., 2006)
		=	PS1	Mouse	Brain	(Chan et al., 2012)
		=	APP_{Sw}	Mouse	Brain	(Chan et al., 2012)
		=	$PS1/APP_{Sw}$	Mouse	Brain	(Chan et al., 2012)
		=	AD	Human	FCx	(Chan et al., 2012)
		=	AD	Human	ECx	(Chan et al., 2012)
		=	AD	Human	Crb	(Chan et al., 2012)
		CON	Wild-type	Rat	Med	(Lohmann et al., 2010)
PC(16:0/20:5)	780	=	PS1	Mouse	Brain	(Chan et al., 2012)
		↑	APP_{Sw}	Mouse	Brain	(Chan et al., 2012)
		↑	$PS1/APP_{Sw}$	Mouse	Brain	(Chan et al., 2012)
		↓	AD	Human	FCx	(Chan et al., 2012)
		=	AD	Human	ECx	(Chan et al., 2012)
		=	AD	Human	Crb	(Chan et al., 2012)
PC(16:0/20:4)	782	↓	$ApoE^{-/-}$	Mouse	SPM	(Igbavboa et al., 2002)
		CON	Wild-type	Mouse	Hip	(Axelsen and Murphy, 2010)
		CON	Wild-type	Mouse	Cx	(Axelsen and Murphy, 2010)
		CON	Wild-type	Rat	SV	(Takamori et al., 2006)

		CON	Wild-type	Rat	Med	(Lohmann et al., 2010)
		CON	Wild-type	Mouse	Hippo.	(Axelsen and Murphy, 2010)
		=	PS1	Mouse	Brain	(Chan et al., 2012)
		↑	APP_{Sw}	Mouse	Brain	(Chan et al., 2012)
		=	$PS1/APP_{Sw}$	Mouse	Brain	(Chan et al., 2012)
		=	AD	Human	FCx	(Chan et al., 2012)
		=	AD	Human	ECx	(Chan et al., 2012)
		=	AD	Human	Crb	(Chan et al., 2012)
PC(16:0/20:3)	784	=	PS1	Mouse	Brain	(Chan et al., 2012)
		=	APP_{Sw}	Mouse	Brain	(Chan et al., 2012)
		=	$PS1/APP_{Sw}$	Mouse	Brain	(Chan et al., 2012)
		=	AD	Human	FCx	(Chan et al., 2012)
		=	AD	Human	ECx	(Chan et al., 2012)
		=	AD	Human	Crb	(Chan et al., 2012)
PC(18:1/18:1)	786	↓	$ApoE^{-/-}$	Mouse	SPM	(Igbavboa et al., 2002)
		=	PS1	Mouse	Brain	(Chan et al., 2012)
		↑	APP_{Sw}	Mouse	Brain	(Chan et al., 2012)
		=	$PS1/APP_{Sw}$	Mouse	Brain	(Chan et al., 2012)
		=	AD	Human	FCx	(Chan et al., 2012)
		=	AD	Human	ECx	(Chan et al., 2012)
		=	AD	Human	Crb	(Chan et al., 2012)
PC(18:0/18:1)	788	=	$ApoE^{-/-}$	Mouse	SPM	(Igbavboa et al., 2002)
		CON	Wild-type	Rat	SV	(Takamori et al., 2006)
		CON	Wild-type	Rat	Med	(Lohmann et al., 2010)
		=	PS1	Mouse	Brain	(Chan et al., 2012)
		↑	APP_{Sw}	Mouse	Brain	(Chan et al., 2012)
		=	$PS1/APP_{Sw}$	Mouse	Brain	(Chan et al., 2012)
		=	AD	Human	FCx	(Chan et al., 2012)
		=	AD	Human	ECx	(Chan et al., 2012)
		=	AD	Human	Crb	(Chan et al., 2012)
PC(18:0/18:0)	790	CON	Wild-type	Mouse	Hip	(Axelsen and Murphy, 2010)
		CON	Wild-type	Mouse	Cx	(Axelsen and Murphy, 2010)
		↑	PS1	Mouse	Brain	(Chan et al., 2012)
		=	APP_{Sw}	Mouse	Brain	(Chan et al., 2012)
		=	$PS1/APP_{Sw}$	Mouse	Brain	(Chan et al., 2012)
		=	AD	Human	FCx	(Chan et al., 2012)
		=	AD	Human	ECx	(Chan et al., 2012)
		=	AD	Human	Crb	(Chan et al., 2012)
PC(16:1/22:6) or PC(18:2/20:5)	804	=	PS1	Mouse	Brain	(Chan et al., 2012)
		=	APP_{Sw}	Mouse	Brain	(Chan et al., 2012)
		=	$PS1/APP_{Sw}$	Mouse	Brain	(Chan et al., 2012)
		=	AD	Human	FCx	(Chan et al., 2012)
		=	AD	Human	ECx	(Chan et al., 2012)
		=	AD	Human	Crb	(Chan et al., 2012)
PC(16:0/22:6)	806	=	PS1	Mouse	Brain	(Chan et al., 2012)
		=	APP_{Sw}	Mouse	Brain	(Chan et al., 2012)
		=	$PS1/APP_{Sw}$	Mouse	Brain	(Chan et al., 2012)
		=	AD	Human	FCx	(Chan et al., 2012)
		=	AD	Human	ECx	(Chan et al., 2012)
		=	AD	Human	Crb	(Chan et al., 2012)
		=	$ApoE^{-/-}$	Mouse	SPM	(Igbavboa et al., 2002)
		CON	Wild-type	Rat	SV	(Takamori et al., 2006)
		CON	Wild-type	Rat	Med	(Lohmann et al., 2010)
PC(16:0/22:5)<br><br>Note: Species PC(38:5) could equally be PC(16:0/22:5) or PC(18:1/20:4).	808	CON	Wild-type	Rat	Med	(Lohmann et al., 2010)
		=	PS1	Mouse	Brain	(Chan et al., 2012)
		↑	APP_{Sw}	Mouse	Brain	(Chan et al., 2012)
		↑	$PS1/APP_{Sw}$	Mouse	Brain	(Chan et al., 2012)
		=	AD	Human	FCx	(Chan et al., 2012)
		=	AD	Human	ECx	(Chan et al., 2012)

		=	AD	Human	Crb	(Chan et al., 2012)
PC(18:1/20:4)	808	CON	Wild-type	Mouse	Hip	(Axelsen and Murphy, 2010)
		CON	Wild-type	Mouse	Cx	(Axelsen and Murphy, 2010)
PC(16:0/22:4)<br><br>Note: Species PC(38:4) could equally be PC(16:0/22:4) or PC(18:0/20:4). Both species are distinguished by Lohmann et al., 2010.	810	CON	Wild-type	Rat	SV	(Takamori et al., 2006)
		=	AD	Human	FCx	(Chan et al., 2012)
		=	AD	Human	ECx	(Chan et al., 2012)
		=	AD	Human	Crb	(Chan et al., 2012)
		=	PS1	Mouse	Brain	(Chan et al., 2012)
		↑	APP_{Sw}	Mouse	Brain	(Chan et al., 2012)
		=	$PS1/APP_{Sw}$	Mouse	Brain	(Chan et al., 2012)
		CON	Wild-type	Rat	Med	(Lohmann et al., 2010)
PC(18:0/20:4)	810	CON	Wild-type	Rat	Med	(Lohmann et al., 2010)
		CON	Wild-type	Mouse	Hip	(Axelsen and Murphy, 2010)
		CON	Wild-type	Mouse	Cx	(Axelsen and Murphy, 2010)
PC(18:0/22:6)	834	CON	Wild-type	Mouse	Hip	(Axelsen and Murphy, 2010)
		=	PS1	Mouse	Brain	(Chan et al., 2012)
		=	APP_{Sw}	Mouse	Brain	(Chan et al., 2012)
		=	$PS1/APP_{Sw}$	Mouse	Brain	(Chan et al., 2012)
		=	AD	Human	FCx	(Chan et al., 2012)
		=	AD	Human	ECx	(Chan et al., 2012)
		=	AD	Human	Crb	(Chan et al., 2012)
PC(18:1/22:6)	832	↓	$ApoE^{-/-}$	Mouse	SPM	(Igbavboa et al., 2002)
		CON	Wild-type	Mouse	Cx	(Axelsen and Murphy, 2010)
		=	PS1	Mouse	Brain	(Chan et al., 2012)
		↑	APP_{Sw}	Mouse	Brain	(Chan et al., 2012)
		=	$PS1/APP_{Sw}$	Mouse	Brain	(Chan et al., 2012)
		=	AD	Human	FCx	(Chan et al., 2012)
		=	AD	Human	ECx	(Chan et al., 2012)
		=	AD	Human	Crb	(Chan et al., 2012)
		CON	Wild-type	Rat	Med	(Lohmann et al., 2010)
PC(18:0/22:5)<br><br>Note: Species PC(40:5) could equally be PC(18:0/22:5) or PC(18:1/22:4).	836	CON	Wild-type	Rat	Med	(Lohmann et al., 2010)
		=	PS1	Mouse	Brain	(Chan et al., 2012)
		=	APP_{Sw}	Mouse	Brain	(Chan et al., 2012)
		=	$PS1/APP_{Sw}$	Mouse	Brain	(Chan et al., 2012)
		=	AD	Human	FCx	(Chan et al., 2012)
		=	AD	Human	ECx	(Chan et al., 2012)
		=	AD	Human	Crb	(Chan et al., 2012)
PC(18:1/22:4)	836	↓	$ApoE^{-/-}$	Mouse	SPM	(Igbavboa et al., 2002)
PC(18:0/22:4)	838	=	$ApoE^{-/-}$	Mouse	SPM	(Igbavboa et al., 2002)
		=	PS1	Mouse	Brain	(Chan et al., 2012)
		=	APP_{Sw}	Mouse	Brain	(Chan et al., 2012)
		=	$PS1/APP_{Sw}$	Mouse	Brain	(Chan et al., 2012)
		=	AD	Human	FCx	(Chan et al., 2012)
		=	AD	Human	ECx	(Chan et al., 2012)
		=	AD	Human	Crb	(Chan et al., 2012)
PC(22:6/22:6)	878	↑	$ApoE^{-/-}$	Mouse	SPM	(Igbavboa et al., 2002)
**PC(*O*-linked) (alkylacylphosphocholines)**						
PC(*O*-12:1/2:0)	466	=	AD	Human	TCx	(Ryan et al., 2009)
PC(*O*-12:0/2:0)	468	=	AD	Human	TCx	(Ryan et al., 2009)
PC(*O*-14:1/2:0)	495	=	AD	Human	TCx	(Ryan et al., 2009)
		CON	Wildtype	Human	Neurons	(Ryan et al., 2009)
PC(*O*-16:0/0:0)	482	↑	AD	Human	TCx	(Ryan et al., 2009)
		CON	Wildtype	Human	Neurons	(Ryan et al., 2009)

PC(*O*-14:0/2:0)	497	=	AD	Human	TCx	(Ryan et al., 2009)
PC(*O*-18:1/0:0)	508	↑	AD	Human	TCx	(Ryan et al., 2009)
		CON	Wildtype	Human	Neurons	(Ryan et al., 2009)
PC(*O*-18:0/0:0)	510	=	AD	Human	TCx	(Ryan et al., 2009)
		CON	Wildtype	Human	Neurons	(Ryan et al., 2009)
PC(*O*-16:3/2:0)	518	=	AD	Human	TCx	(Ryan et al., 2009)
PC(*O*-16:2/2:0)	520	=	AD	Human	TCx	(Ryan et al., 2009)
PC(*O*-16:1/2:0)	522	=	AD	Human	TCx	(Ryan et al., 2009)
		CON	Wildtype	Human	Neurons	(Ryan et al., 2009)
PC(*O*-16:0/2:0)	524	↑	AD	Human	TCx	(Ryan et al., 2009)
		↑	TgCRND8	Mouse	TCx	(Ryan et al., 2009)
		CON	Wildtype	Human	Neurons	(Ryan et al., 2009)
PC(*O*-20:0/2:0)	538	CON	Wildtype	Human	Neurons	(Ryan et al., 2009)
PC(*O*-18:4/2:0)	544	=	AD	Human	TCx	(Ryan et al., 2009)
		CON	Wildtype	Human	Neurons	(Ryan et al., 2009)
PC(*O*-18:3/2:0)	546	=	AD	Human	TCx	(Ryan et al., 2009)
PC(*O*-18:2/2:0)	548	=	AD	Human	TCx	(Ryan et al., 2009)
PC(*O*-18:1/2:0)	550	=	AD	Human	TCx	(Ryan et al., 2009)
PC(*O*-18:0/2:0)	550	=	AD	Human	TCx	(Ryan et al., 2009)
		CON	Wildtype	Human	Neurons	(Ryan et al., 2009)
PC(*O*-20:6/2:0)	570	=	AD	Human	TCx	(Ryan et al., 2009)
		CON	Wildtype	Human	Neurons	(Ryan et al., 2009)
PC(*O*-20:4/2:0)	574	=	AD	Human	TCx	(Ryan et al., 2009)
		CON	Wildtype	Human	Neurons	(Ryan et al., 2009)
PC(*O*-20:0/2:0)	580	=	AD	Human	TCx	(Ryan et al., 2009)

[a] Stereospecificity of *sn*-1 and *sn*-2 chains was assigned by the authors based on (1) the reported total carbon number and total degree of unsaturation provided in the original datasets (i.e., (Takamori et al., 2006; Chan et al., 2012)) and (2) the most likely isobaric species present in neural cells and brain tissue established (a) empirically in the datasets using standard addition or analysis of *lyso*-form fragment ions attributed to the neutral loss of fatty acyl moieties using MS^2 or MS^3 spectra or (b) predicted in published literature (i.e., (Igbavboa et al., 2002; Whitehead et al., 2007; Smith et al., 2008; Ryan et al., 2009; Hou et al., 2011). In cases where predominant species have yet to be identified empirically or where multiple isobaric species are known to be present in neural membranes, all possible choices are indicated curating for chain length and degree of saturation and stereospecificity considered most likely to appear in mammalian cellular membranes based on prevalence (Miyazaki and Ntambi, 2008) and as predicted using VaLID v1.0.1 (Blanchard et al., 2013).

[b] m/z is reported for [M-H]+ ions (phosphocholines) or [M-H]- ions (all others).

[c] =, ↓,↑ indicate comparisons relative to appropriate controls. CON indicates control data only (i.e., identified in control tissue but not compared to another condition). ND indicates not detected.

[d] First value summarizes changes at 2 months; second value changes at 12 months.

[e] First value summarizes changes in grey matter, second value changes in white matter (of the same patient).

[f] First value summarizes changes at 9 months, second value changes at 18 months.

[g] Datasets:

(1) Profile of isolated rat synaptic vesicles (Takamori et al., 2006);

(2) Profile WT rat hippocampus (Axelsen and Murphy, 2010);

(3) Profile of WT mouse cortex (Axelsen and Murphy, 2010);

(4) - DHA Depletion: The effects of embryonic or postnatal dietary depletion of the DHA precursor α-linolenic acid (18:3n-3) was assessed at postnatal day 1 (neonates) and 1 month old (postnatal) Wistar rats compared to controls fed an adequate diet (Brand et al., 2010);

(5) Comparison of apoliprotein E null mutants ($ApoE^{-/-}$) with wild-type (WT) C57BL/6J mice, 2-3 months of age (Igbavboa et al., 2002);

(6-10) Comparison of APP_{sw} mice transgenic for human APP with the Swedish double mutation K670N/M671L aged between 9-11.5 months of age (onset) with WT mice (Chan et al., 2012); Comparison of this same mouse model at 9 months (onset) and 18 months (symptomatic) of age (Han et al., 2001);

(11) Comparison of mice transgenic for PS1 aged between 9-11.5 months (symptomatic) with WT mice (Chan et al., 2012);

(12) Comparison of double transgenic mice APP_{sw} x PS1 aged between 9-11.5 months (symptomatic) with WT mice (Chan et al., 2012);

(13-23) Comparison of Alzheimer Disease patient with age- and gender-matched controls postmortem in various brain regions (Han et al., 2001; Ryan et al., 2009; Chan et al., 2012);

(24) Comparison of $Ctsd^{-/-}$ cathepsin D null mutant mice with WT controls (Mutka et al., 2010);

(25) Comparison of ApoE ε2, ApoE ε3, and ApoE ε4 knockin of human ApoE variants into the murine ApoE locus (humanized mouse model) with congenic N8 C57Bl/6 x 129P2 mice at 2 (young) and 12 (middle-aged) months of age

(Sharman et al., 2010);

(26) Profile of rat Medulla (Lohmann et al., 2010);

(27-28) Comparison of TgCRND8 mice transgenic for human APP with both Swedish (K670N/M671L) and Indiana mutations (V717F) with congenic N4 C57Bl/6 x C3H littermates at both 2 (pre-symptomatic) and 4 (onset) months of age;

(29) Profile of WT mouse cerebrum/brain (Eberlin et al., 2010);

[h] Tissue: Brain, Cerebrum; Cx, Cortex; Crb, cerebellum; ECx, Entorhinal Cortex; FCx, Frontal Cortex/Prefrontal Cortex; Hip, Hippocampus; PCx, Parietal Cortex; SPM, Synaptosomal membranes; SV, Synaptic Vesicle.

Supplemental Table 3: Changes in phosphoserine composition in neural tissue, synaptic membranes, and synaptic vesicles of postmortem human AD patients and experimental models of AD and AD risk compared to normal elderly (human) or congenic controls (animals models): A comparison of 12 independent neurolipidomic datasets from seven different laboratories

Phosphoserines

Molecular Species[a]	m/z[b]	Relative abundance[c]	Datasets[g]	Cohort	Source[h]	References
**Total PS (diacylphosphatidylserines)**		↑	$ApoE^{-/-}$	Mouse	SPM	(Igbavboa et al., 2002)
		↓ = [d]	ApoE ε2 KI	Mouse	Brain	(Sharman et al., 2010)
		= = [d]	ApoE ε3 KI	Mouse	Brain	(Sharman et al., 2010)
		= = [d]	ApoE ε4 KI	Mouse	Brain	(Sharman et al., 2010)
		↓	PS1	Mouse	Brain	(Chan et al., 2012)
		↓	APP_{Sw}	Mouse	Brain	(Chan et al., 2012)
		↓	PS1/APP_{Sw}	Mouse	Brain	(Chan et al., 2012)
		=	AD	Human	FCx	(Chan et al., 2012)
		=	AD	Human	ECx	(Chan et al., 2012)
		=	AD	Human	Crb	(Chan et al., 2012)
PS(16:0/0:0)	496	=	PS1	Mouse	Brain	(Chan et al., 2012)
		=	APP_{Sw}	Mouse	Brain	(Chan et al., 2012)
		=	PS1/ APP_{Sw}	Mouse	Brain	(Chan et al., 2012)
		=	AD	Human	FCx	(Chan et al., 2012)
		=	AD	Human	ECx	(Chan et al., 2012)
		=	AD	Human	Crb	(Chan et al., 2012)
PS(18:1/0:0)	522	=	PS1	Mouse	Brain	(Chan et al., 2012)
		=	APP_{Sw}	Mouse	Brain	(Chan et al., 2012)
		=	PS1/ APP_{Sw}	Mouse	Brain	(Chan et al., 2012)
		=	AD	Human	FCx	(Chan et al., 2012)
		=	AD	Human	ECx	(Chan et al., 2012)
		=	AD	Human	Crb	(Chan et al., 2012)
PS (18:0/0:0)	524	=	PS1	Mouse	Brain	(Chan et al., 2012)
		=	APP_{Sw}	Mouse	Brain	(Chan et al., 2012)
		=	PS1/ APP_{Sw}	Mouse	Brain	(Chan et al., 2012)
		=	AD	Human	FCx	(Chan et al., 2012)
		=	AD	Human	ECx	(Chan et al., 2012)
		=	AD	Human	Crb	(Chan et al., 2012)
PS(16:0/16:0)	734	CON	- DHA	Rat	Cx	(Brand et al., 2010)
PS(16:0/18:2)	758	↓	PS1	Mouse	Brain	(Chan et al., 2012)
		↓	APP_{Sw}	Mouse	Brain	(Chan et al., 2012)
		↓	PS1/APP_{Sw}	Mouse	Brain	(Chan et al., 2012)
		=	AD	Human	FCx	(Chan et al., 2012)
		=	AD	Human	ECx	(Chan et al., 2012)
		=	AD	Human	Crb	(Chan et al., 2012)
PS(16:0/18:1)	760	↑	$ApoE^{-/-}$	Mouse	SPM	(Igbavboa et al., 2002)
		↓	PS1	Mouse	Brain	(Chan et al., 2012)
		↓	APP_{Sw}	Mouse	Brain	(Chan et al., 2012)
		↓	PS1/APP_{Sw}	Mouse	Brain	(Chan et al., 2012)
		=	AD	Human	FCx	(Chan et al., 2012)
		=	AD	Human	ECx	(Chan et al., 2012)
		=	AD	Human	Crb	(Chan et al., 2012)
PS(16:0/20:4)	782	=	$ApoE^{-/-}$	Mouse	SPM	(Igbavboa et al., 2002)
		↓	PS1	Mouse	Brain	(Chan et al., 2012)
		↓	APP_{Sw}	Mouse	Brain	(Chan et al., 2012)
		↓	PS1/APP_{Sw}	Mouse	Brain	(Chan et al., 2012)
		=	AD	Human	FCx	(Chan et al., 2012)
		=	AD	Human	ECx	(Chan et al., 2012)

		=	AD	Human	Crb	(Chan et al., 2012)
PS(16:0/20:3)	784	↓	PS1	Mouse	Brain	(Chan et al., 2012)
		↓	APP_{Sw}	Mouse	Brain	(Chan et al., 2012)
		↓	PS1/APP_{Sw}	Mouse	Brain	(Chan et al., 2012)
		=	AD	Human	FCx	(Chan et al., 2012)
		=	AD	Human	ECx	(Chan et al., 2012)
		=	AD	Human	Crb	(Chan et al., 2012)
PS(18:1/18:1)	786	↓	PS1	Mouse	Brain	(Chan et al., 2012)
		↓	APP_{Sw}	Mouse	Brain	(Chan et al., 2012)
		↓	PS1/APP_{Sw}	Mouse	Brain	(Chan et al., 2012)
		=	AD	Human	FCx	(Chan et al., 2012)
		=	AD	Human	ECx	(Chan et al., 2012)
		=	AD	Human	Crb	(Chan et al., 2012)
PS(18:0/18:1)	788	=	$ApoE^{-/-}$	Mouse	SPM	(Igbavboa et al., 2002)
		=	PS1	Mouse	Brain	(Chan et al., 2012)
		↓	APP_{Sw}	Mouse	Brain	(Chan et al., 2012)
		=	PS1/APP_{Sw}	Mouse	Brain	(Chan et al., 2012)
		=	AD	Human	FCx	(Chan et al., 2012)
		=	AD	Human	ECx	(Chan et al., 2012)
		=	AD	Human	Crb	(Chan et al., 2012)
PS(18:0/18:0)	790	CON	- DHA	Rat	Cx	(Brand et al., 2010)
		CON	Wild-type	Mouse	Hip	(Axelsen and Murphy, 2010)
		CON	Wild-type	Mouse	Cx	(Axelsen and Murphy, 2010)
PS(16:0/22:6)	806	=	$ApoE^{-/-}$	Mouse	SPM	(Igbavboa et al., 2002)
PS(16:0/22:5)	808	↓	$ApoE^{-/-}$	Mouse	SPM	(Igbavboa et al., 2002)
		↓	PS1	Mouse	Brain	(Chan et al., 2012)
		↓	APP_{Sw}	Mouse	Brain	(Chan et al., 2012)
		↓	PS1/APP_{Sw}	Mouse	Brain	(Chan et al., 2012)
		=	AD	Human	FCx	(Chan et al., 2012)
		=	AD	Human	ECx	(Chan et al., 2012)
		=	AD	Human	Crb	(Chan et al., 2012)
PS(16:0/22:4)<br><br>Note: Species PS(38:4) could equally be PS(16:0/22:4) or PS(18:0/20:4). Both species are distinguished by Igbavboa et al., 2002.	810	=	$ApoE^{-/-}$	Mouse	SPM	(Igbavboa et al., 2002)
		CON	- DHA	Rat	Cx	(Brand et al., 2010)
		↓	PS1	Mouse	Brain	(Chan et al., 2012)
		↓	APP_{Sw}	Mouse	Brain	(Chan et al., 2012)
		↓	PS1/APP_{Sw}	Mouse	Brain	(Chan et al., 2012)
		=	AD	Human	FCx	(Chan et al., 2012)
		=	AD	Human	ECx	(Chan et al., 2012)
		=	AD	Human	Crb	(Chan et al., 2012)
PS(18:0/20:4)	810	↑	$ApoE^{-/-}$	Mouse	SPM	(Igbavboa et al., 2002)
		CON	Wild-type	Mouse	Hip	(Axelsen and Murphy, 2010)
		CON	Wild-type	Mouse	Cx	(Axelsen and Murphy, 2010)
PS(18:0/20:3)	812	↓	PS1	Mouse	Brain	(Chan et al., 2012)
		↓	APP_{Sw}	Mouse	Brain	(Chan et al., 2012)
		↓	PS1/APP_{Sw}	Mouse	Brain	(Chan et al., 2012)
		=	AD	Human	FCx	(Chan et al., 2012)
		=	AD	Human	ECx	(Chan et al., 2012)
		=	AD	Human	Crb	(Chan et al., 2012)
PS(16:0/22:2)<br>or PS(18:0/20:4)	814	↓	PS1	Mouse	Brain	(Chan et al., 2012)
		↓	APP_{Sw}	Mouse	Brain	(Chan et al., 2012)
		=	PS1/APP_{Sw}	Mouse	Brain	(Chan et al., 2012)
		=	AD	Human	FCx	(Chan et al., 2012)
		=	AD	Human	ECx	(Chan et al., 2012)
		=	AD	Human	Crb	(Chan et al., 2012)
PS(18:1/22:6)	832	=	$ApoE^{-/-}$	Mouse	SPM	(Igbavboa et al., 2002)
		↓	PS1	Mouse	Brain	(Chan et al., 2012)
		↓	APP_{Sw}	Mouse	Brain	(Chan et al., 2012)

		↓	PS1/APP_{Sw}	Mouse	Brain	(Chan et al., 2012)
		=	AD	Human	FCx	(Chan et al., 2012)
		=	AD	Human	ECx	(Chan et al., 2012)
		=	AD	Human	Crb	(Chan et al., 2012)
PS(18:0/22:6)	835	↓	$ApoE^{-/-}$	Mouse	SPM	(Igbavboa et al., 2002)
		CON	Wild-type	Mouse	Brain	(Eberlin et al., 2010)
		CON[j]	- DHA	Rat	Cx	(Brand et al., 2010)
		CON[j]	Wild-type	Rat	SV	(Takamori et al., 2006)
		CON	Wild-type	Mouse	Hip	(Axelsen and Murphy, 2010)
		CON	Wild-type	Mouse	Cx	(Axelsen and Murphy, 2010)
		↓	PS1	Mouse	Brain	(Chan et al., 2012)
		↓	APP_{Sw})	Mouse	Brain	(Chan et al., 2012)
		↓	PS1/APP_{Sw}	Mouse	Brain	(Chan et al., 2012)
		=	AD	Human	FCx	(Chan et al., 2012)
		=	AD	Human	ECx	(Chan et al., 2012)
		=	AD	Human	Crb	(Chan et al., 2012)
PS(20:0/20:5)	836	↓	PS1	Mouse	Brain	(Chan et al., 2012)
		↓	APP_{Sw}	Mouse	Brain	(Chan et al., 2012)
		↓	PS1/APP_{Sw}	Mouse	Brain	(Chan et al., 2012)
		=	AD	Human	FCx	(Chan et al., 2012)
		=	AD	Human	ECx	(Chan et al., 2012)
		=	AD	Human	Crb	(Chan et al., 2012)
PS(18:0/22:4)	838	↓	$ApoE^{-/-}$	Mouse	SPM	(Igbavboa et al., 2002)
		CON	- DHA	Rat	Cx	(Brand et al., 2010)
		CON	Wild-type	Rat	SV	(Takamori et al., 2006)
		=	PS1	Mouse	Brain	(Chan et al., 2012)
		=	APP_{Sw}	Mouse	Brain	(Chan et al., 2012)
		=	PS1/APP_{Sw}	Mouse	Brain	(Chan et al., 2012)
		=	AD	Human	FCx	(Chan et al., 2012)
		=	AD	Human	ECx	(Chan et al., 2012)
		↓	AD	Human	Crb	(Chan et al., 2012)
PS(22:6/22:6)	878	↑	$ApoE^{-/-}$	Mouse	SPM	(Igbavboa et al., 2002)
PS(22:5/22:6)	880	↓	$ApoE^{-/-}$	Mouse	SPM	(Igbavboa et al., 2002)
PS(22:4/22:6)	882	↑	$ApoE^{-/-}$	Mouse	SPM	(Igbavboa et al., 2002)

[a] Stereospecificity of *sn*-1 and *sn*-2 chains was assigned by the authors based on (1) the reported total carbon number and total degree of unsaturation provided in the original datasets (i.e., (Takamori et al., 2006; Chan et al., 2012)) and (2) the most likely isobaric species present in neural cells and brain tissue established (a) empirically in the datasets using standard addition or analysis of *lyso*-form fragment ions attributed to the neutral loss of fatty acyl moieties using MS^2 or MS^3 spectra or (b) predicted in published literature (i.e., (Igbavboa et al., 2002; Whitehead et al., 2007; Smith et al., 2008; Ryan et al., 2009; Hou et al., 2011). In cases where predominant species have yet to be identified empirically or where multiple isobaric species are known to be present in neural membranes, all possible choices are indicated curating for chain length and degree of saturation and stereospecificity considered most likely to appear in mammalian cellular membranes based on prevalence (Miyazaki and Ntambi, 2008) and as predicted using VaLID v1.0.1 (Blanchard et al., 2013).

[b] m/z is reported for [M-H]+ ions (phosphocholines) or [M-H]- ions (all others).

[c] =, ↓,↑ indicate comparisons relative to appropriate controls. CON indicates control data only (i.e., identified in control tissue but not compared to another condition). ND indicates not detected.

[d] First value summarizes changes at 2 months; second value changes at 12 months.

[e] First value summarizes changes in grey matter, second value changes in white matter (of the same patient).

[f] First value summarizes changes at 9 months, second value changes at 18 months.

[g] Datasets:

(1) Profile of isolated rat synaptic vesicles (Takamori et al., 2006);

(2) Profile WT rat hippocampus (Axelsen and Murphy, 2010);

(3) Profile of WT mouse cortex (Axelsen and Murphy, 2010);

(4) - DHA Depletion: The effects of embryonic or postnatal dietary depletion of the DHA precursor α-linolenic acid (18:3n-3) was assessed at postnatal day 1 (neonates) and 1 month old (postnatal) Wistar rats compared to controls fed an adequate diet (Brand et al., 2010);

(5) Comparison of apoliprotein E null mutants ($ApoE^{-/-}$) with wild-type (WT) C57BL/6J mice, 2-3 months of age (Igbavboa et al., 2002);

(6-10) Comparison of APP_{sw} mice transgenic for human APP with the Swedish double mutation K670N/M671L aged between 9-11.5 months of age (onset) with WT mice (Chan et al., 2012); Comparison of this same mouse model at 9 months (onset) and 18 months (symptomatic) of age (Han et al., 2001);

(11) Comparison of mice transgenic for PS1 aged between 9-11.5 months (symptomatic) with WT mice (Chan et al., 2012);

(12) Comparison of double transgenic mice APP_{sw} x PS1 aged between 9-11.5 months (symptomatic) with WT mice (Chan et al., 2012);

(13-23) Comparison of Alzheimer Disease patient with age- and gender-matched controls postmortem in various brain regions (Han et al., 2001; Ryan et al., 2009; Chan et al., 2012);

(24) Comparison of $Ctsd^{-/-}$ cathepsin D null mutant mice with WT controls (Mutka et al., 2010);

(25) Comparison of ApoE ε2, ApoE ε3, and ApoE ε4 knockin of human ApoE variants into the murine ApoE locus (humanized mouse model) with congenic N8 C57Bl/6 x 129P2 mice at 2 (young) and 12 (middle-aged) months of age (Sharman et al., 2010);

(26) Profile of rat Medulla (Lohmann et al., 2010);

(27-28) Comparison of TgCRND8 mice transgenic for human APP with both Swedish (K670N/M671L) and Indiana mutations (V717F) with congenic N4 C57Bl/6 x C3H littermates at both 2 (pre-symptomatic) and 4 (onset) months of age;

(29) Profile of WT mouse cerebrum/brain (Eberlin et al., 2010);

[h] Tissue: Brain, Cerebrum; Cx, Cortex; Crb, cerebellum; ECx, Entorhinal Cortex; FCx, Frontal Cortex/Prefrontal Cortex; Hip, Hippocampus; PCx, Parietal Cortex; SPM, Synaptosomal membranes; SV, Synaptic Vesicle.

Supplemental Table 4: Changes in phosphoinositol composition in neural tissue, synaptic membranes, and synaptic vesicles of postmortem human AD patients and experimental models of AD and AD risk compared to normal elderly (human) or congenic controls (animals models): A comparison of ten independent neurolipidomic datasets from five different laboratories

Phosphoinositols

Molecular Species[a]	m/z[b]	Relative abundance[c]	Datasets[g]	Cohort	Source[h]	References
**Total PI (diacylphosphatidylinositols)**		=	$ApoE^{-/-}$	Mouse	SPM	(Igbavboa et al., 2002)
		= =[d]	ApoE ε2 KI	Mouse	Brain	(Sharman et al., 2010)
		= =[d]	ApoE ε3 KI	Mouse	Brain	(Sharman et al., 2010)
		= =[d]	ApoE ε4 KI	Mouse	Brain	(Sharman et al., 2010)
PI(16:0/18:1)	835	=	$ApoE^{-/-}$	Mouse	SPM	(Igbavboa et al., 2002)
		↓	PS1	Mouse	Brain	(Chan et al., 2012)
		↓	APP_{Sw}	Mouse	Brain	(Chan et al., 2012)
		↓	PS1/APP_{Sw}	Mouse	Brain	(Chan et al., 2012)
		=	AD	Human	FCx	(Chan et al., 2012)
		=	AD	Human	ECx	(Chan et al., 2012)
		=	AD	Human	Crb	(Chan et al., 2012)
PI(16:0/20:4)	857	↓	$ApoE^{-/-}$	Mouse	SPM	(Igbavboa et al., 2002)
		↓	PS1	Mouse	Brain	(Chan et al., 2012)
		↓	APP_{Sw}	Mouse	Brain	(Chan et al., 2012)
		↓	PS1/APP_{Sw}	Mouse	Brain	(Chan et al., 2012)
		=	AD	Human	FCx	(Chan et al., 2012)
		=	AD	Human	ECx	(Chan et al., 2012)
		=	AD	Human	Crb	(Chan et al., 2012)
		CON	- DHA	Rat	Cx	(Brand et al., 2010)
PI(18:0/18:1)	863	=	$ApoE^{-/-}$	Mouse	SPM	(Igbavboa et al., 2002)
		=	AD	Human	FCx	(Chan et al., 2012)
		=	AD	Human	ECx	(Chan et al., 2012)
		=	AD	Human	Crb	(Chan et al., 2012)
		↓	PS1	Mouse	Brain	(Chan et al., 2012)
		↓	APP_{Sw}	Mouse	Brain	(Chan et al., 2012)
		↓	PS1/APP_{Sw}	Mouse	Brain	(Chan et al., 2012)
PI(16:0/22:6)	881	↓	$ApoE^{-/-}$	Mouse	SPM	(Igbavboa et al., 2002)
		↓	PS1	Mouse	Brain	(Chan et al., 2012)
		↓	APP_{Sw}	Mouse	Brain	(Chan et al., 2012)
		↓	PS1/APP_{Sw}	Mouse	Brain	(Chan et al., 2012)
		=	AD	Human	FCx	(Chan et al., 2012)
		=	AD	Human	ECx	(Chan et al., 2012)
		=	AD	Human	Crb	(Chan et al., 2012)
PI(18:1/20:4)	883	CON	- DHA	Rat	Cx	(Brand et al., 2010)
		↓	PS1	Mouse	Brain	(Chan et al., 2012)
		↓	APP_{Sw}	Mouse	Brain	(Chan et al., 2012)
		↓	PS1/APP_{Sw}	Mouse	Brain	(Chan et al., 2012)
		=	AD	Human	FCx	(Chan et al., 2012)
		=	AD	Human	ECx	(Chan et al., 2012)
		=	AD	Human	Crb	(Chan et al., 2012)
PI(18:0/20:4)	885	↑	$ApoE^{-/-}$	Mouse	SPM	(Igbavboa et al., 2002)
		↓	PS1	Mouse	Brain	(Chan et al., 2012)
		↓	APP_{Sw}	Mouse	Brain	(Chan et al., 2012)
		↓	PS1/APP_{Sw}	Mouse	Brain	(Chan et al., 2012)

		=	AD	Human	FCx	(Chan et al., 2012)
		=	AD	Human	ECx	(Chan et al., 2012)
		=	AD	Human	Crb	(Chan et al., 2012)
		CON	- DHA	Rat	Cx	(Brand et al., 2010)
		CON	Wild-type	Mouse	Hip	(Axelsen and Murphy, 2010)
		CON	Wild-type	Mouse	Cx	(Axelsen and Murphy, 2010)
PI(18:0/22:6)	909	↓	$ApoE^{-/-}$	Mouse	SPM	(Igbavboa et al., 2002)
		↓	PS1	Mouse	Brain	(Chan et al., 2012)
		↓	APP_{Sw}	Mouse	Brain	(Chan et al., 2012)
		↓	PS1/APP_{Sw}	Mouse	Brain	(Chan et al., 2012)
		=	AD	Human	FCx	(Chan et al., 2012)
		=	AD	Human	ECx	(Chan et al., 2012)
		=	AD	Human	Crb	(Chan et al., 2012)

[a] Stereospecificity of *sn*-1 and *sn*-2 chains was assigned by the authors based on (1) the reported total carbon number and total degree of unsaturation provided in the original datasets (i.e., (Takamori et al., 2006; Chan et al., 2012)) and (2) the most likely isobaric species present in neural cells and brain tissue established (a) empirically in the datasets using standard addition or analysis of *lyso*-form fragment ions attributed to the neutral loss of fatty acyl moieties using MS^2 or MS^3 spectra or (b) predicted in published literature (i.e., (Igbavboa et al., 2002; Whitehead et al., 2007; Smith et al., 2008; Ryan et al., 2009; Hou et al., 2011). In cases where predominant species have yet to be identified empirically or where multiple isobaric species are known to be present in neural membranes, all possible choices are indicated curating for chain length and degree of saturation and stereospecificity considered most likely to appear in mammalian cellular membranes based on prevalence (Miyazaki and Ntambi, 2008) and as predicted using VaLID v1.0.1 (Blanchard et al., 2013).

[b] m/z is reported for [M-H]+ ions (phosphocholines) or [M-H]- ions (all others).

[c] =, ↓,↑ indicate comparisons relative to appropriate controls. CON indicates control data only (i.e., identified in control tissue but not compared to another condition). ND indicates not detected.

[d] First value summarizes changes at 2 months; second value changes at 12 months.

[e] First value summarizes changes in grey matter, second value changes in white matter (of the same patient).

[f] First value summarizes changes at 9 months, second value changes at 18 months.

[g] Datasets:

(1) Profile of isolated rat synaptic vesicles (Takamori et al., 2006);
(2) Profile WT rat hippocampus (Axelsen and Murphy, 2010);
(3) Profile of WT mouse cortex (Axelsen and Murphy, 2010);
(4) - DHA Depletion: The effects of embryonic or postnatal dietary depletion of the DHA precursor α-linolenic acid (18:3n-3) was assessed at postnatal day 1 (neonates) and 1 month old (postnatal) Wistar rats compared to controls fed an adequate diet (Brand et al., 2010);
(5) Comparison of apoliprotein E null mutants ($ApoE^{-/-}$) with wild-type (WT) C57BL/6J mice, 2-3 months of age (Igbavboa et al., 2002);
(6-10) Comparison of APP_{sw} mice transgenic for human APP with the Swedish double mutation K670N/M671L aged between 9-11.5 months of age (onset) with WT mice (Chan et al., 2012); Comparison of this same mouse model at 9 months (onset) and 18 months (symptomatic) of age (Han et al., 2001);
(11) Comparison of mice transgenic for PS1 aged between 9-11.5 months (symptomatic) with WT mice (Chan et al., 2012);
(12) Comparison of double transgenic mice APP_{sw} x PS1 aged between 9-11.5 months (symptomatic) with WT mice (Chan et al., 2012);
(13-23) Comparison of Alzheimer Disease patient with age- and gender-matched controls postmortem in various brain regions (Han et al., 2001; Ryan et al., 2009; Chan et al., 2012);
(24) Comparison of $Ctsd^{-/-}$ cathepsin D null mutant mice with WT controls (Mutka et al., 2010);
(25) Comparison of ApoE ε2, ApoE ε3, and ApoE ε4 knockin of human ApoE variants into the murine ApoE locus (humanized mouse model) with congenic N8 C57Bl/6 x 129P2 mice at 2 (young) and 12 (middle-aged) months of age (Sharman et al., 2010);
(26) Profile of rat Medulla (Lohmann et al., 2010);
(27-28) Comparison of TgCRND8 mice transgenic for human APP with both Swedish (K670N/M671L) and Indiana mutations (V717F) with congenic N4 C57Bl/6 x C3H littermates at both 2 (pre-symptomatic) and 4 (onset) months of age;
(29) Profile of WT mouse cerebrum/brain (Eberlin et al., 2010);

[h] Tissue: Brain, Cerebrum; Cx, Cortex; Crb, cerebellum; ECx, Entorhinal Cortex; FCx, Frontal Cortex/Prefrontal Cortex; Hip, Hippocampus; PCx, Parietal Cortex; SPM, Synaptosomal membranes; SV, Synaptic Vesicle.

Supplemental Table 5: Changes in free fatty acids, neuroprotectins, and prostaglandins in neural tissue of postmortem human AD patients and experimental models of AD compared to normal elderly (human) or congenic controls (animals models): A comparison of four independent neurolipidomic datasets from four different laboratories

PLA_2- liberated bioactive free fatty acids and some downstream metabolites

Family	Molecular Species	Relative abundance[a]	Datasets[b]	Cohort	Source[c]	References
Bioactive fatty acids	Total Free Fatty Acids	↑	J20 APP_{FAD}	Mouse	Hip	(Sanchez-Mejia et al., 2008)
		=	J20 APP_{FAD}	Mouse	Cx	(Sanchez-Mejia et al., 2008)
		=	I5 APP_{WT}	Mouse	Hip	(Sanchez-Mejia et al., 2008)
	Monounsaturated free fatty acids	↑	AD	Human	Hip	(Astarita et al., 2011)
		↑	AD	Human	FCx	(Astarita et al., 2011)
		=	AD	Human	TCx	(Astarita et al., 2011)
		=	AD	Human	Crb	(Astarita et al., 2011)
	Saturated free fatty acids	=	AD	Human	Hip	(Astarita et al., 2011)
		=	AD	Human	FCx	(Astarita et al., 2011)
		=	AD	Human	TCx	(Astarita et al., 2011)
		=	AD	Human	Crb	(Astarita et al., 2011)
	Arachidic acid (20:0)	↑	AD	Human	Hip	(Astarita et al., 2011)
		=	AD	Human	FCx	(Astarita et al., 2011)
		=	AD	Human	TCx	(Astarita et al., 2011)
		=	AD	Human	Crb	(Astarita et al., 2011)
	Arachidonic acid (20:4)	↑	J20 APP_{FAD}	Mouse	Cx	(Sanchez-Mejia et al., 2008)
		=	I5 APP_{WT}	Mouse	Hip	(Sanchez-Mejia et al., 2008)
		↓	$cPLA_2^{-/-}$ X J20 APP_{FAD}	Mouse	Hip	(Sanchez-Mejia et al., 2008)
		=	J20 APP_{FAD}	Mouse	Hip	(Sanchez-Mejia et al., 2008)
		=	AD	Human	FCx	(Fraser et al., 2010)
		=	AD	Human	TCx	(Fraser et al., 2010)
		=	AD	Human	PCx	(Fraser et al., 2010)
	Behenic acid (22:0)	↑	AD	Human	Hip	(Astarita et al., 2011)
		=	AD	Human	FCx	(Astarita et al., 2011)
		=	AD	Human	TCx	(Astarita et al., 2011)
		=	AD	Human	Crb	(Astarita et al., 2011)
	Docosahexaenoic acid (22:6)	↓	AD	Human	Hip	(Lukiw et al., 2005)
		=	AD	Human	FCx	(Fraser et al., 2010)
		↓	AD	Human	TCx	(Lukiw et al., 2005)
		=	AD	Human	TCx	(Fraser et al., 2010)
		=	AD	Human	PCx	(Fraser et al., 2010)
		↓	AD	Human	OCx	(Lukiw et al., 2005)
		↓	AD	Human	Thal	(Lukiw et al., 2005)
	Eicosapentaenoic Acid (20:5)	=	J20 APP_{FAD}	Mouse	Hip	(Sanchez-Mejia et al., 2008)
	Eicosenoic acid (20:1)	↑	AD	Human	Hip	(Astarita et al., 2011)
		↑	AD	Human	FCx	(Astarita et al., 2011)
		=	AD	Human	FCx	(Fraser et al., 2010)
		=	AD	Human	TCx	(Astarita et al., 2011)
		=	AD	Human	TCx	(Fraser et al., 2010)
		=	AD	Human	PCx	(Fraser et al., 2010)
		=	AD	Human	Crb	(Astarita et al., 2011)
	Erucic acid (22:1)	=	AD	Human	Hip	(Astarita et al., 2011)
		↑	AD	Human	FCx	(Astarita et al., 2011)
		=	AD	Human	TCx	(Astarita et al., 2011)
		=	AD	Human	Crb	(Astarita et al., 2011)
	Hexacosenoic acid (26:1)	=	AD	Human	Hip	(Astarita et al., 2011)
		↑	AD	Human	FCx	(Astarita et al., 2011)

		↑	AD	Human	TCx	(Astarita et al., 2011)
		=	AD	Human	Crb	(Astarita et al., 2011)
	Linoleic acid	=	J20 APP_{FAD}	Mouse	Hip	(Sanchez-Mejia et al., 2008)
		=	AD	Human	FCx	(Fraser et al., 2010)
		=	AD	Human	TCx	(Fraser et al., 2010)
		=	AD	Human	PCx	(Fraser et al., 2010)
	α-linolenic acid	=	J20 APP_{FAD}	Mouse	Hip	(Sanchez-Mejia et al., 2008)
	Mead acid (20:3)	↑	AD	Human	Hip	(Astarita et al., 2011)
		↑	AD	Human	FCx	(Astarita et al., 2011)
		↑	AD	Human	TCx	(Astarita et al., 2011)
		=	AD	Human	Crb	(Astarita et al., 2011)
	Myristic acid (14:0)	=	AD	Human	FCx	(Fraser et al., 2010)
		=	AD	Human	TCx	(Fraser et al., 2010)
			AD	Human	PCx	(Fraser et al., 2010)
	Nervonic acid (24:1)	=	AD	Human	Hip	(Astarita et al., 2011)
		↑	AD	Human	FCx	(Astarita et al., 2011)
		↑	AD	Human	TCx	(Astarita et al., 2011)
		=	AD	Human	Crb	(Astarita et al., 2011)
	Oleic acid (18:1 n-9)	↑	AD	Human	Hip	(Astarita et al., 2011)
		↑	AD	Human	FCx	(Astarita et al., 2011)
		↑	AD	Human	FCx	(Fraser et al., 2010)
		=	AD	Human	TCx	(Astarita et al., 2011)
		↑	AD	Human	TCx	(Fraser et al., 2010)
		=	AD	Human	PCx	(Fraser et al., 2010)
		=	AD	Human	Crb	(Astarita et al., 2011)
	Palmitic acid (16:0)	=	AD	Human	Hip	(Astarita et al., 2011)
		=	AD	Human	FCx	(Astarita et al., 2011)
		=	AD	Human	FCx	(Fraser et al., 2010)
		=	AD	Human	TCx	(Astarita et al., 2011)
		=	AD	Human	TCx	(Fraser et al., 2010)
		↑	AD	Human	PCx	(Fraser et al., 2010)
		=	AD	Human	Crb	(Astarita et al., 2011)
	Palmitoleic acid (16:1)	↑	AD	Human	Hip	(Astarita et al., 2011)
		↑	AD	Human	FCx	(Astarita et al., 2011)
		=	AD	Human	FCx	(Fraser et al., 2010)
		=	AD	Human	TCx	(Astarita et al., 2011)
		=	AD	Human	TCx	(Fraser et al., 2010)
		=	AD	Human	PCx	(Fraser et al., 2010)
		=	AD	Human	Crb	(Astarita et al., 2011)
	Stearic acid (18:0)	=	AD	Human	Hip	(Astarita et al., 2011)
		=	AD	Human	FCx	(Astarita et al., 2011)
		↓	AD	Human	FCx	(Fraser et al., 2010)
		=	AD	Human	TCx	(Astarita et al., 2011)
		↓	AD	Human	TCx	(Fraser et al., 2010)
		=	AD	Human	PCx	(Fraser et al., 2010)
		=	AD	Human	Crb	(Astarita et al., 2011)
**NeuroprotectinD1**	Neuroprotectin D1	↓	AD	Human	Hip	(Lukiw et al., 2005)
		↓	AD	Human	TCx	(Lukiw et al., 2005)
		=	AD	Human	OCx	(Lukiw et al., 2005)
		=	AD	Human	Thal	(Lukiw et al., 2005)
**Prostaglandins**	PGE_2 (and PGB_2 degradation product)	↑	J20	Mouse	Hip	(Sanchez-Mejia et al., 2008)
		=	J20	Mouse	Cx	(Sanchez-Mejia et al., 2008)

[a]=, ↓,↑ indicate comparisons relative to appropriate controls. ND indicates not detected.
[b] Datasets:
(1) Comparison of J20 mice transgenic for human APP with both Swedish (K670N/M671L) and Indiana familial mutations (V717F), I5 mice transgenic for human WT APP , or J20 X $cPLA_2^{-/-}$ with congenic C57Bl/6 NonTg littermates (4 months of age) (Sanchez-Mejia et al., 2008)

(2) Comparison of Alzheimer Disease patient with age- and gender-matched controls postmortem in various brain regions (Lukiw et al., 2005; Fraser et al., 2010; Astarita et al., 2011)

[c]Tissue: Cx, Cortex; Crb, cerebellum; FCx, Frontal Cortex/Prefrontal Cortex; Hip, Hippocampus; OCx, Occipital Cortex; PCx, Parietal Cortex; TCx, Temporal Cortex; Thal, thalamus.
